# Intakes of Added Sugars, with a Focus on Beverages and the Associations with Micronutrient Adequacy in US Children, Adolescents, and Teens (NHANES 2003–2018)

**DOI:** 10.3390/nu15153285

**Published:** 2023-07-25

**Authors:** Laurie Ricciuto, Victor L. Fulgoni, P. Courtney Gaine, Maria O. Scott, Loretta DiFrancesco

**Affiliations:** 1 Department of Nutritional Sciences, University of Toronto, Toronto, ON M5S 1A1, Canada; 2 Nutrition Impact, LLC, Battle Creek, MI 49014, USA; vic3rd@aol.com; 3 The Sugar Association, Inc., Washington, DC 20005, USA; gaine@sugar.org (P.C.G.); mscott@sugar.org (M.O.S.); 4 Source! Nutrition, Toronto, ON M6S 5A6, Canada; loretta@sourcenutrition.com

**Keywords:** added sugars, sweetened beverages, micronutrient intake, micronutrient adequacy, children, adolescents, US, NHANES

## Abstract

Added sugars intake from sweetened beverages among children, adolescents, and teens is a public health concern. This study examined the relationships between added sugars intake from specific types of beverages with added sugars and from the rest of the diet (excluding beverages with added sugars) and micronutrient adequacy among US children, adolescents, and teens. Data from eight consecutive 2 y cycles of NHANES were combined (2003–04 through 2017–18), and regression analysis was conducted to test for trends in quantiles of added sugars intake from each beverage source (soft drinks, fruit drinks, sport and energy drinks, coffee and tea, and flavored milk) and the rest of the diet (excluding those beverages) and micronutrient adequacy among children (2–8 y) and adolescents and teens (9–18 y). Among those aged 2–8 y, higher added sugars from flavored milk were associated with lower percentages below the estimated average requirement (EAR) for calcium. Among those aged 9–18 y, higher added sugars from soft drinks or coffee and tea were associated with higher percentages below the EAR for magnesium and vitamins A and C. In contrast, higher added sugars from fruit drinks or flavored milk were associated with lower percentages below the EAR (higher percentages above the adequate intake (AI)) for vitamin C (fruit drinks) and calcium, magnesium, phosphorus, vitamin A, and potassium (flavored milk). Regarding the rest of the diet, higher added sugars were associated with lower percentages below the EAR (higher percentages above the AI) for most micronutrients examined. The results suggest that the relationship between added sugars intake and micronutrient adequacy depends on the added sugar sources and their nutrient composition. Continued monitoring of sweetened beverage consumption, including beverage type, and the association with added sugars intake, micronutrient adequacy, and diet quality is warranted, given the changes in consumption and product development over time.

## 1. Introduction

The 2020–2025 Dietary Guidelines for Americans (DGA) have established recommendations for healthy eating patterns for optimal health for the US population [1]. Adherence to the DGA recommendations varies across the lifespan; the diets of children (2–8 y) tend to be more closely aligned with the recommendations compared to other age groups, while the diets of adolescents and teens (9–18 y) tend to depart the most [1]. In particular, excess consumption of added sugars (≥10% energy per day) and inadequate intakes of calcium, vitamin D, and potassium are public health concerns for both age groups; also, those aged 9–18 y have intakes below the recommendations for magnesium, phosphorus, and choline [1]. Improving dietary shortfalls and excesses among children, adolescents, and teens is important for their healthy growth and development and to help reduce the risk of chronic disease later in life [1].

Regarding the consumption of added sugars, intakes of sweetened beverages (mainly soft drinks and fruit drinks) are the main concern, as these beverages are the top contributors to added sugars intake among children, adolescents, and teens [2,3,4,5]. Recent estimates from US national survey data for 2017–18 indicate that soft drinks and fruit drinks together accounted for 21% of total daily added sugars intake among children (2–8 y) and 30% among adolescents and teens (9–18 y) [2]. Research also shows that fruit drinks are the top contributor to added sugars intake among children, while soft drinks are the top contributor among adolescents and teens [2,6]. Other beverages contributing to added sugars intake among children, adolescents, and teens include flavored milk and pre-sweetened coffee and tea (hereafter, coffee and tea), with variations depending on age. In 2017–18, added sugars from flavored milk accounted for 6% of total daily added sugars intake among those aged 2–8 y [2], while added sugars from coffee and tea accounted for 7% of total daily intake among those aged 9–18 y [4], with contributions from flavored milk declining with age and contributions from coffee and tea increasing [6,7].

The consumption of beverages with added sugars is a concern among children, adolescents, and teens, given the research showing inverse associations with diet quality [8,9,10,11] and micronutrient intakes [7,11,12]. Most studies have examined “sugar-sweetened beverages” (SSBs) and have defined them in various ways, typically including soft drinks, fruit drinks, sport and energy drinks, and coffee and tea; in some studies, sweetened water [8,9,10] and/or flavored milk [11] have also been included. Regardless of how SSBs are defined, studies have consistently demonstrated inverse associations between these beverages and diet quality and micronutrient intakes, with higher SSB consumption associated with lower intakes of milk, dairy, vegetables, and fruit [10,11], lower scores on a composite diet quality index (i.e., Healthy Eating Index, HEI) [8,9,10], and lower intakes of micronutrients, such as calcium, magnesium, phosphorus, vitamins A and D, and potassium [7,11,12]. In contrast, some beverages with added sugars have been shown to contribute positively to micronutrient intakes among children, adolescents, and teens [6,12,13,14]; in particular, the consumption of fruit drinks can contribute to vitamin C intake [14], and the consumption of flavored milk can contribute to the intakes of calcium [6,13], magnesium, phosphorus, vitamin D, and potassium [6].

The potential opposite associations between different types of beverages with added sugars and micronutrient intakes are important considerations with regard to micronutrient adequacy. While there is evidence that higher intakes of added sugars are associated with greater percentages of children, adolescents, and teens with intakes below the EAR for some micronutrients [15] and there are some inverse associations between SSB consumption and micronutrient intake [7,11,12], there is a paucity of research examining the associations between added sugars intake and micronutrient adequacy at the level of specific types of beverages with added sugars. Examining the associations between added sugars intake and micronutrient adequacy at a more granular level could help to inform dietary guidance and policies focused on reducing the consumption of specific beverages as a means to reduce the excess consumption of added sugars without compromising micronutrient intakes.

Furthermore, while beverages with added sugars are top contributors to added sugars intake among children, adolescents, and teens, foods also contribute to added sugars intake. However, studies typically examine SSBs in isolation, and research on added sugars and micronutrient intakes examining both food and beverage sources of added sugars is limited [11]. A more fulsome examination of the associations between added sugars intake and micronutrient adequacy would align well with the total diet approach of the DGA.

The purpose of the present study was to examine the relationships between added sugars intake from specific types of beverages with added sugars (soft drinks, fruit drinks, sport and energy drinks, coffee and tea, and flavored milk) and micronutrient adequacy for the nutrients of public health concern among US children, adolescents, and teens; and, for context, to examine the relationship between added sugars intake from the rest of the diet (excluding beverages with added sugars) and micronutrient adequacy.

## 2. Materials and Methods

### 2.1. Data

To examine the relationship between added sugars from specific beverages and micronutrient adequacy among children, adolescents, and teens (2–18 y), data from eight consecutive 2 y cycles of NHANES were combined (2003–04 through 2017–18 cycles). Diet and health are monitored regularly in the US through NHANES, a nationally representative cross-sectional survey of non-institutionalized civilian residents conducted by the US National Center for Health Statistics (NCHS), which is part of the Centers for Disease Control and Prevention (CDC); details on the survey design, data collection, and analytic procedures are reported elsewhere [16,17]. The dietary interview component of NHANES, called What We Eat in America (WWEIA), consists of two non-consecutive 24 h recalls using the five-step Automated Multiple-Pass Method administered by trained interviewers [18]. Interviews are conducted with a proxy for children aged 2–5 y and are proxy-assisted for children aged 6–11 y; NHANES obtains written, informed consent for all participants. Only subjects with 2 d of dietary recall data were included in the analyses to enable the calculation of usual intakes required for the assessment of micronutrient adequacy. Of those aged 2–18 y, *n* = 7164 were excluded due to missing or unreliable dietary data, and *n* = 77 pregnant or lactating females were also excluded, resulting in a final analytic sample size of *n* = 21,005, with *n* = 8599 children (2–8 y) and *n* = 12,406 adolescents and teens (9–18 y) (Supplemental Figure S1).

### 2.2. Added Sugars Intake

The mean added sugars intakes from beverages with added sugars were determined using the USDA Food Patterns and Equivalents Database (FPED) specific to each NHANES cycle and the average of both dietary recalls. The FPED converts food and beverage intakes into food group equivalents corresponding to those in the DGA [19]. The added sugars food pattern component is comprised of caloric sweeteners using the definition of “sugars that are added to foods as an ingredient during preparation, processing or at the table; and do not include naturally occurring sugars, such as lactose present in milk and fructose present in whole or cut fruit and 100% fruit juice” [19]. The beverages with added sugars we analyzed were based on the 2017–18 WWEIA food categories, in which foods and beverages are grouped by the USDA based on similar nutrient content and common use in the diet [20]. The WWEIA food categories include individual categories for soft drinks, fruit drinks, sport and energy drinks, coffee and tea, and flavored milk, allowing us to examine these beverages separately. In addition, we calculated the mean added sugars intake from the WWEIA food category “sweetened beverages”, which combines soft drinks, fruit drinks, sport and energy drinks, nutritional beverages, smoothies, and grain drinks. For context, we also calculated the mean added sugars intake from the rest of the diet, which comprised all foods and beverages except the beverages with added sugars that we examined (i.e., the WWEIA sweetened beverage group, coffee and tea, and flavored milk) (Table 1).

### 2.3. Added Sugars Intake and Micronutrient Adequacy

The added sugars intake was calculated as the percentage of the total daily calories (% kcal) to account for differences in energy intake over time. To examine the relationships between the added sugars intake from the various beverages with added sugars and micronutrient adequacy, the mean added sugars intakes within each beverage group were divided into quantiles. For the added sugars intakes from each of the beverage groups, non-consumers comprised the first quantile, and the consumers were divided into tertiles (quantiles 2, 3, and 4). For the added sugars intake from the rest of the diet, given the very low number of non-consumers (<0.5% of the population), quartiles of the added sugars intake were established.

The intakes of micronutrients previously reported to be under-consumed among children, namely calcium, magnesium, phosphorus, vitamins A, C, and D, choline, and potassium [1,21], were obtained from the NHANES dietary intake files. To estimate the usual intake (UI) and distribution of intakes of micronutrients for each age group, the National Cancer Institute (NCI) method was used [22]. Given that most micronutrients were consumed on most days by most subjects, the one-part model was used for the UI estimations. The 2 d of intake, using 2 d sampling weights, were used to obtain the percentiles of intake and necessary variance estimates. The covariates used in the NCI UI estimations were the day of the week of the 24 h recall (coded as the weekend (Friday to Sunday) or weekday (Monday to Thursday)) and the sequence of dietary recall (first or second). Balanced repeated replication was performed to generate standard errors; balanced repeated replication weights were generated using a Fay adjustment factor of M = 0.3 with a perturbation factor of 0.7, which were then adjusted to match the initial sample weights within the age, sex, and race and ethnicity groups. The EAR cut-point method [23] was used to estimate the percentage of an age group with intakes below the requirements within each quantile of added sugars intake for each beverage source and for the rest of the diet. For the micronutrients with an AI, namely choline and potassium, we determined the percentage of individuals consuming greater than the AI. Micronutrient intakes from dietary supplements were not included because the primary research question focused on intakes from beverages with added sugars and from the rest of the diet.

### 2.4. Statistical Analyses

The data were analyzed using SAS 9.4 (SAS Institute, Cary, NC, USA). Dietary sample weights provided by NHANES were used to adjust for the complex survey sampling design, design changes across survey cycles, non-response rates, and oversampling of certain subgroups. Regression analysis was used to examine the associations between the quantiles of added sugars intakes from beverages with added sugars and from the rest of the diet and the % <EAR (or % >AI) for the selected micronutrients. Within each beverage source, linear trends in the % <EAR (or % >AI) across the quantiles of added sugars intake (quantiles 1–4) were assessed. Additionally, the difference in the % <EAR between the non-consumers (quantile 1) and consumers (quantiles 2, 3, and 4) was assessed, and given the large number of non-consumers for many of the beverage sources, the linear trends across the tertiles of consumers only (quantiles 2–4) were also assessed. Furthermore, for the beverages in which changes in consumption occurred over time, trend analyses were repeated in 4 y cycles for the combined age group (2–18 y) for a sufficient sample size. A value of *p* < 0.01 was deemed statistically significant; however, differences that were *p* < 0.05 and had an effect size considered nutritionally significant (a difference of 5% <EAR or >AI) were also highlighted.

## 3. Results

### 3.1. Consumption Patterns of Beverage Sources of Added Sugars and Added Sugars Intake

The vast majority (68.6–79.1%) of children, adolescents, and teens reported consuming a sweetened beverage in the previous 24 h, i.e., consumers (Table 2). Within the WWEIA sweetened beverages category, soft drinks and fruit drinks had the highest levels of reporting, ranging from 36.2–58.2%. For the other two beverage categories, flavored milk had a higher level of reporting compared to coffee and tea, especially among those aged 2–8 y, with three times more consumers of flavored milk than coffee and tea. Considering the variations by age, there were more consumers of fruit drinks and flavored milk among those aged 2–8 y compared to those aged 9–18 y, while there were more consumers of soft drinks, sport and energy drinks, and coffee and tea among those aged 9–18 y compared to those aged 2–8 y.

The mean daily energy intake was 1680 ± 8 kcal among those aged 2–8 y, with 237 ± 2 kcal coming from added sugars; and 2037 ± 13 kcal among those aged 9–18 y, with 331 ± 4 kcal coming from added sugars. Added sugars (% kcal) from soft drinks, fruit drinks, sport and energy drinks, coffee and tea, and flavored milk accounted for approximately one-third of the total added sugars intake among those aged 2–8 y, while it accounted for approximately one-half among those aged 9–18 y; and added sugars (% kcal) from the rest of the diet accounted for approximately two-thirds of the total intake among those aged 2–8 y and one-half among those aged 9–18 y (Table 3). Among the consumers aged 2–8 y, soft drinks and fruit drinks each accounted for approximately 30% of the total added sugars intake, while sport and energy drinks, coffee and tea, and flavored milk each accounted for approximately 20% of the total added sugars intake. Among the consumers aged 9–18 y, soft drinks accounted for approximately 45% of the total added sugars intake, while fruit drinks, sport and energy drinks, and coffee and tea each accounted for approximately 28% of the total added sugars intake (Table 3).

**Figure 1:**
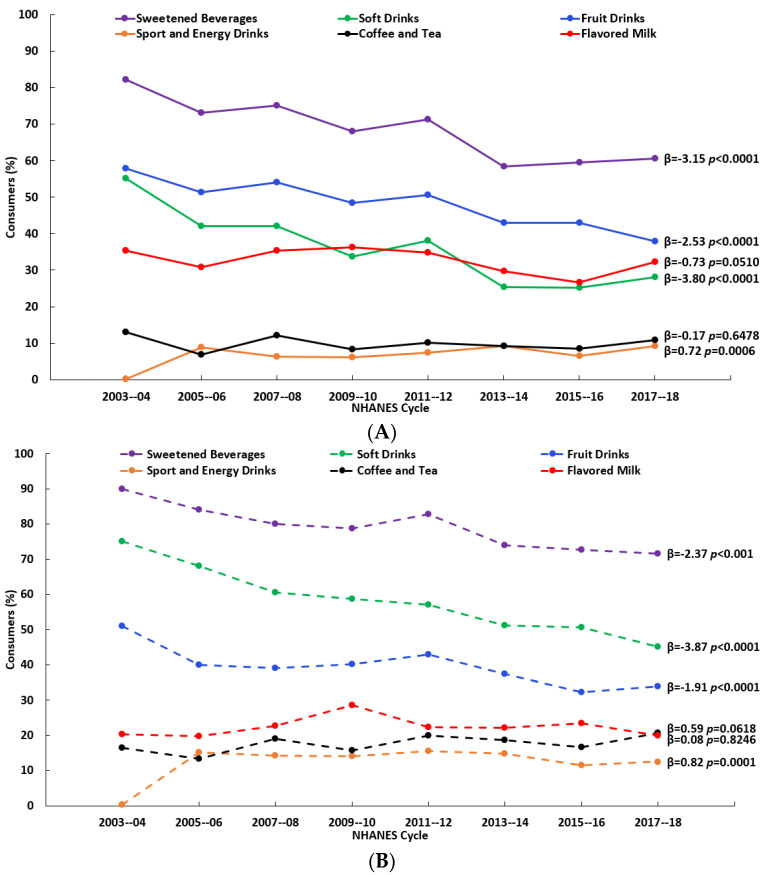
Percentages of those reporting intake (consumers) of beverage sources of added sugars by NHANES cycle among (**A**) children (2–8 y, *n* = 8599) and (**B**) adolescents and teens (9–18 y, *n* = 12,406); β and *p*-values from linear trend analysis, significant at *p* < 0.01; source NHANES 2003–04 to 2017–18.

**Figure 2:**
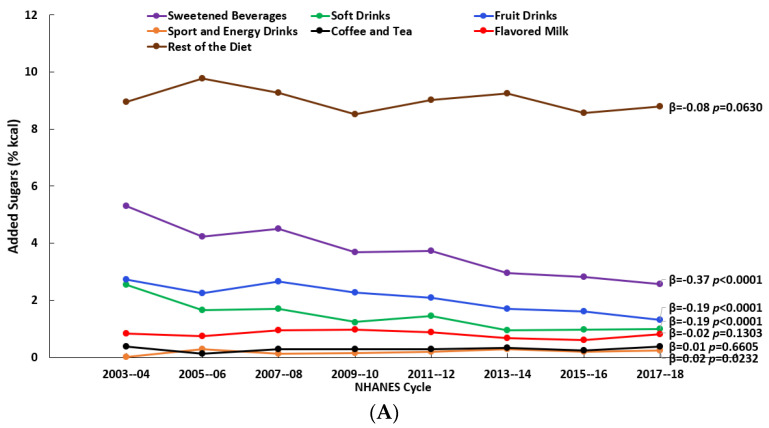

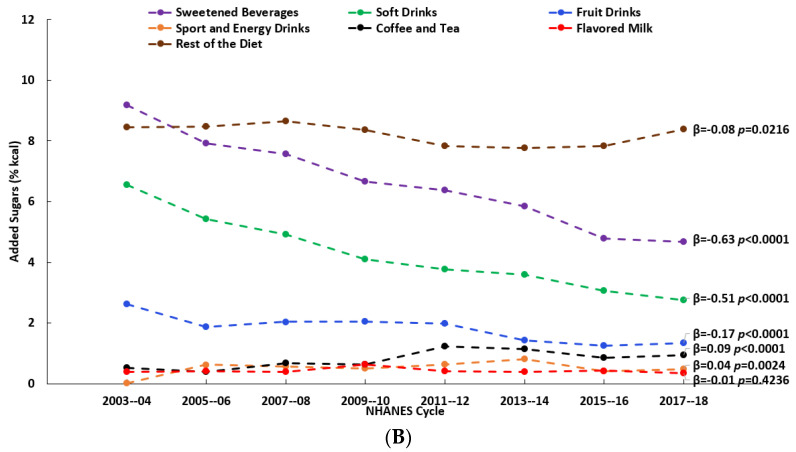
Added sugars intake (% kcal) from beverage sources and the rest of the diet by NHANES cycle among all individuals: (**A**) children (2–8 y) and (**B**) adolescents and teens (9–18 y); β and *p*-values from linear trend analysis, significant at *p* < 0.01; source NHANES 2003–04 to 2017–18.

**Figure 3:**
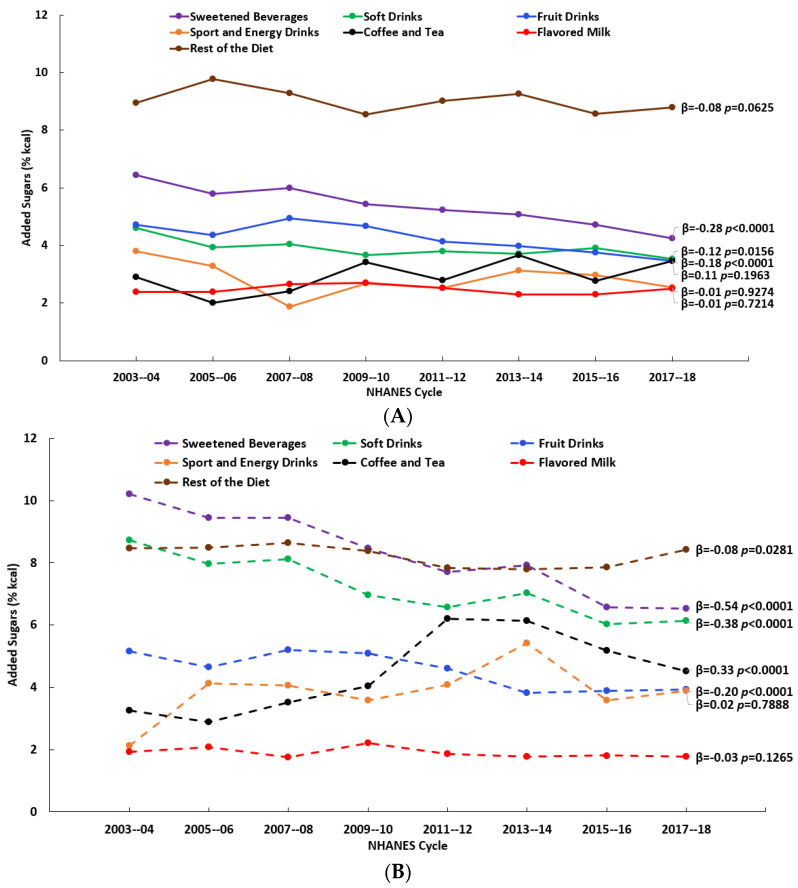
Added sugars intake (% kcal) from beverage sources and the rest of the diet by NHANES cycle among consumers: (**A**) children (2–8 y) and (**B**) adolescents and teens (9–18 y); β and p-values from linear trend analysis, significant at *p* < 0.01; source NHANES 2003–04 to 2017–18.

The consumption of soft drinks, fruit drinks, sport and energy drinks, and coffee and tea changed (significantly at *p* < 0.01) over the 16-y time span (2003–2018) to varying degrees and with variations by age. For both age groups (2–8 y and 9–18 y), there were decreases in the percentages who reported consuming soft drinks and fruit drinks and an increase in the percentage who reported consuming sport and energy drinks (Figure 1A,B, Supplemental Table S1). Added sugars (% kcal) from soft drinks and fruit drinks decreased over time for both age groups, while added sugars from sport and energy drinks and coffee and tea increased over time, but only among those aged 9–18 y (Figure 2A,B, Supplemental Table S2). Considering the consumers only, added sugars from fruit drinks decreased among those aged 2–8 y, while among those aged 9–18 y, there were decreases in added sugars from both soft drinks and fruit drinks and an increase in those from coffee and tea (Figure 3A,B, Supplemental Table S2). The patterns for the beverages with added sugars were similar for the added sugars intake in grams (Supplemental Table S3). There were no changes over time in added sugars from the rest of the diet when expressed as % kcal (Figure 2A,B, Supplemental Table S2), but there were significant decreases (*p* < 0.01) when they were expressed in grams (Supplemental Table S3).

### 3.2. Associations between Added Sugars Intake and Micronutrient Adequacy by Beverage Source

Among the consumers of each type of beverage, the tertile ranges of added sugars intake varied by type of beverage and by age. The lowest added sugars intakes were from flavored milk among those aged 9–18 y, with a range across the tertiles from 1.1 to >2.0% kcal, while the highest added sugars intakes were from soft drinks among those aged 9–18 y, with a range across the tertiles from 4.0 to >7.9% kcal (Table 4).

Children (2–8 y): Significant relationships between the added sugars intake and micronutrient adequacy emerged for three beverage sources—soft drinks, fruit drinks, and flavored milk (Table 5). The percentages of individuals with potassium intakes above the AI was significantly lower (*p* < 0.01) among the consumers of soft drinks compared to the non-consumers, though this difference was small, at 3.3% units. Likewise, the percentage of individuals with vitamin C intakes below the EAR was significantly lower (*p* < 0.01) among the consumers of fruit drinks compared to the non-consumers, but by a difference of only 1.7% units. There was a bigger difference for vitamin D, in which the percentage of individuals with intakes below the EAR was significantly higher (*p* = 0.02), by 5.4% units for fruit drink consumers versus non-consumers. For flavored milk, significant (*p* < 0.01) but small effect sizes were detected for phosphorus and vitamin A, with lower percentages of children with intakes below the EAR (0.1% units and 3.9% units, respectively) among the consumers versus the non-consumers. Additionally, for flavored milk, there was a significant graded relationship (*p* < 0.01) for calcium, with a decrease of 7.4% units in the percentage of individuals with intakes below the EAR with each increase in quantile of added sugars, and a larger 9.3% unit decrease when consumers only were included in the analysis.

Adolescents and teens (9–18 y): Significant relationships between the added sugars intake and micronutrient adequacy emerged for the WWEIA sweetened beverages group and for four specific beverage sources—soft drinks, fruit drinks, coffee and tea, and flavored milk (Table 6). For sweetened beverages (WWEIA), only vitamin C was significantly associated (*p* = 0.02) with added sugars intake, with the percentage of individuals with vitamin C intakes below the EAR being 11.5% units lower among the consumers versus the non-consumers. For soft drinks, higher added sugars intake was significantly associated with greater percentages of individuals with intakes below the EAR for magnesium (5.7% unit increase with each quantile, *p* < 0.01; 6.9% units among consumers only, *p* < 0.01), vitamin A (6.8% unit increase with each quantile, *p* = 0.01), and vitamin C (6.2% unit increase with each quantile, *p* = 0.02). Additionally, compared to the non-consumers of soft drinks, the percentage of individuals with vitamin D intakes below the EAR was significantly higher (*p* < 0.01) among the consumers, by a difference of 2.7% units, and the percentage with potassium intakes above the AI was significantly lower (*p* = 0.01) to a larger degree, at 8.7% units. For fruit drinks, higher added sugars intake was associated with significantly lower (*p* = 0.03) percentages of individuals with vitamin C intakes below the EAR, a 15.6% unit decrease with each quantile. Additionally, compared to the non-consumers of fruit drinks, the percentages of individuals with magnesium and vitamin C intakes below the EAR were significantly lower (*p* = 0.04) among the consumers by 7.6% units and 36.8% units, respectively, and the percentage with vitamin D intakes below the EAR was significantly higher (*p* < 0.01), but to a smaller degree, at 2.2% units. For coffee and tea, a higher added sugars intake was associated with significantly higher (*p* = 0.02) percentages of individuals with vitamin C intakes below the EAR, an increase of 6.3% units for each quantile. For flavored milk, a higher added sugars intake was associated with significantly lower percentages of individuals with intakes below the EAR for calcium (*p* = 0.02), magnesium (*p* = 0.03), phosphorus (*p* < 0.01), and vitamin A (*p* = 0.04), and an intake of potassium above the AI (*p* = 0.04), ranging in magnitude from 8.8% units to 14.4% units. There were also significant differences between the consumers and non-consumers of flavored milk in percentages below the EAR for magnesium (21.7% units higher, *p* = 0.02), phosphorus (20.9% units higher, *p* = 0.05), and vitamin A (26.5% units higher, *p* = 0.01).

The associations of added sugars with micronutrient adequacy for the combined 2–18 y age group are available in Supplemental Table S4.

**Table 6:** Trends in % <EAR (>AI ^1^) for selected micronutrients by beverage source of added sugars among adolescents and teens (9–18 y, *n* = 12,406) from the pooled sample (NHANES 2003–2018, *n* = 21,005); weighted n’s are shown in the table.

Sweetened Beverages							
	Q1 ^2^ *n* = 8,714,811 % <EAR (SE)	Q2 *n* = 11,013,572 % <EAR (SE)	Q3 *n* = 11,011,003 % <EAR (SE)	Q4 *n* = 11,028,124 % <EAR (SE)	Quantile Trend ^3^ Beta (SE), *p*	Q1 vs. Q2, 3, 4 ^4^ Beta (SE), *p*	Q2–Q4 Trend ^5^ Beta (SE), *p*
Calcium	62.09 (2.32)	62.63 (1.86)	68.26 (1.91)	64.16 (2.03)	1.07 (1.39), 0.52	2.96 (3.95), 0.53	0.66 (2.81), 0.85
Magnesium	55.50 (2.13)	52.72 (1.65)	61.35 (1.48)	66.17 (1.30)	4.42 (1.61), 0.11	4.87 (9.27), 0.65	6.68 (1.10), 0.10
Phosphorus	26.99 (2.12)	25.86 (1.63)	28.53 (1.69)	19.01 (1.79)	−2.37 (1.89), 0.34	−2.67 (6.85), 0.73	−3.56 (3.52), 0.50
Vitamin A	32.06 (2.35)	31.46 (1.89)	43.79 (1.84)	49.95 (2.36)	6.94 (1.73), 0.06	10.07 (12.80), 0.51	9.18 (1.78), 0.12
Vitamin C	40.05 (2.41)	27.56 (2.25)	30.03 (2.31)	28.01 (2.58)	−2.88 (2.15), 0.31	−11.51 (1.80), 0.02 ^§^	0.18 (1.30), 0.91
Vitamin D	92.21 (1.08)	93.89 (0.86)	96.12 (0.67)	96.39 (0.58)	1.44 (0.31), 0.04	3.31 (1.85), 0.22	1.23 (0.57), 0.27
Choline ^1^	6.01 (1.17)	3.72 (0.70)	3.13 (0.67)	3.15 (0.64)	−0.86 (0.38), 0.15	−2.67 (0.44), 0.03	−0.29 (0.18), 0.35
Potassium ^1^	32.41 (2.15)	28.25 (2.04)	25.52 (1.76)	29.54 (1.77)	−0.85 (1.42), 0.61	−4.62 (2.84), 0.25	0.72 (1.95), 0.78
**Soft Drinks**							
	**Q1 ^2^** ***n* = 17,449,876** **% <EAR (SE)**	**Q2** ***n* = 8,034,425** **% <EAR (SE)**	**Q3** ***n* = 8,161,395** **% <EAR (SE)**	**Q4** ***n* = 8,121,813** **% <EAR (SE)**	**Quantile Trend ^3^** **Beta (SE),** ***p***	**Q1 vs. Q2, 3, 4 ^4^** **Beta (SE),** ***p***	**Q2–Q4 Trend ^5^** **Beta (SE),** ***p***
Calcium	61.68 (1.51)	66.03 (2.33)	68.35 (2.43)	62.56 (2.31)	0.88 (1.50), 0.62	3.95 (2.66), 0.28	−1.73 (2.32), 0.59
Magnesium	52.91 (1.22)	56.57 (2.01)	63.47 (1.47)	70.28 (1.56)	5.73 (0.51), 0.01 ^†^	10.50 (6.29), 0.24	6.85 (0.03), 0.00 ^†^
Phosphorus	25.92 (1.36)	28.86 (1.70)	25.31 (2.23)	18.36 (2.12)	−2.17 (1.46), 0.28	−1.73 (4.90), 0.76	−5.25 (0.97), 0.12
Vitamin A	32.15 (1.63)	36.61 (2.23)	47.01 (2.31)	51.74 (2.65)	6.80 (0.72), 0.01 ^§^	12.92 (7.11), 0.21	7.57 (1.62), 0.13
Vitamin C	24.55 (1.63)	28.31 (2.36)	34.30 (2.89)	44.14 (3.31)	6.23 (0.88), 0.02 ^§^	11.01 (7.33), 0.27	7.91 (1.10), 0.09
Vitamin D	93.18 (0.61)	95.85 (0.89)	96.10 (0.79)	95.66 (0.83)	0.92 (0.47), 0.19	2.69 (0.20), 0.01 ^†^	−0.09 (0.20), 0.73
Choline ^1^	4.92 (0.70)	3.74 (0.88)	2.52 (0.79)	3.35 (0.95)	−0.69 (0.31), 0.15	−1.71 (0.56), 0.09	−0.23 (0.60), 0.77
Potassium ^1^	33.80 (1.54)	24.35 (2.20)	24.48 (1.80)	26.42 (2.25)	−2.76 (1.76), 0.26	−8.72 (1.06), 0.01 ^§^	1.03 (0.51), 0.29
**Fruit Drinks**							
	**Q1 ^2^** ***n* = 25,242,572** **% <EAR (SE)**	**Q2** ***n* = 5,506,152** **% <EAR (SE)**	**Q3** ***n* = 5,508,295** **% <EAR (SE)**	**Q4** ***n* = 5,510,490** **% <EAR (SE)**	**Quantile Trend ^3^** **Beta (SE),** ***p***	**Q1 vs. Q2, 3, 4 ^4^** **Beta (SE),** ***p***	**Q2–Q4 Trend ^5^** **Beta (SE),** ***p***
Calcium	63.35 (1.44)	67.43 (2.48)	66.09 (2.35)	62.85 (2.54)	0.18 (0.96), 0.87	1.87 (1.89), 0.43	−2.34 (0.55), 0.15
Magnesium	61.84 (1.10)	52.35 (2.15)	56.40 (2.08)	53.88 (1.77)	−2.69 (1.38), 0.19	−7.57 (1.55), 0.04 ^§^	0.60 (1.88), 0.80
Phosphorus	25.24 (1.21)	28.11 (2.41)	25.81 (2.67)	19.79 (2.18)	−1.34 (1.14), 0.36	−1.10 (3.45), 0.78	−4.25 (1.07), 0.16
Vitamin A	40.39 (1.45)	36.02 (2.61)	36.59 (2.25)	42.68 (2.82)	0.16 (1.34), 0.92	−1.61 (2.99), 0.65	3.47 (1.58), 0.27
Vitamin C	46.02 (1.72)	20.59 (2.94)	6.96 (2.63)	2.76 (1.77)	−15.58 (2.73), 0.03 ^§^	−36.80 (7.14), 0.04 ^§^	−8.68 (2.70), 0.19
Vitamin D	93.83 (0.59)	96.22 (0.83)	95.97 (0.83)	96.00 (0.83)	0.81 (0.34), 0.14	2.23 (0.10), 0.00 ^†^	−0.10 (0.08), 0.44
Choline ^1^	4.39 (0.63)	3.17 (0.97)	3.16 (0.64)	4.31 (0.97)	−0.18 (0.31), 0.61	−0.81 (0.52), 0.26	0.58 (0.33), 0.33
Potassium ^1^	27.44 (1.32)	25.66 (2.28)	30.27 (2.63)	37.41 (2.36)	2.89 (1.17), 0.13	4.28 (4.70), 0.46	5.94 (0.72), 0.08
**Sport and Energy Drinks**							
	**Q1 ^2^** ***n* = 36,649,246** **% <EAR (SE)**	**Q2** ***n* = 1,670,286** **% <EAR (SE)**	**Q3** ***n* = 1,716,552** **% <EAR (SE)**	**Q4** ***n* = 1,978,772** **% <EAR (SE)**	**Quantile Trend ^3^** **Beta (SE),** ***p***	**Q1 vs. Q2, 3, 4 ^4^** **Beta (SE),** ***p***	**Q2–Q4 Trend ^5^** **Beta (SE),** ***p***
Calcium	64.95 (1.16)	68.08 (4.46)	60.40 (6.06)	46.78 (5.51)	−4.30 (2.04), 0.17	−5.92 (6.56), 0.46	−10.57 (1.69), 0.10
Magnesium	58.63 (0.89)	63.98 (3.67)	62.81 (3.64)	58.90 (5.06)	1.05 (1.14), 0.46	3.40 (1.61), 0.17	−2.50 (0.78), 0.19
Phosphorus	25.25 (1.04)	32.42 (4.72)	20.91 (5.05)	18.47 (3.55)	−1.55 (1.91), 0.50	−0.86 (4.63), 0.87	−7.10 (2.58), 0.22
Vitamin A	39.49 (1.24)	42.73 (4.10)	44.41 (5.60)	36.24 (7.14)	0.25 (1.33), 0.87	1.78 (2.57), 0.56	−3.10 (2.81), 0.47
Vitamin C	32.14 (1.37)	14.00 (6.58)	29.00 (4.85)	22.26 (9.02)	−3.87 (3.18), 0.35	−10.73 (4.67), 0.15	4.44 (6.19), 0.60
Vitamin D	94.74 (0.44)	96.17 (1.65)	95.42 (1.98)	92.82 (2.63)	−0.21 (0.48), 0.71	0.16 (1.06), 0.89	−1.65 (0.52), 0.20
Choline ^1^	3.97 (0.46)	3.31 (1.51)	3.58 (1.65)	6.39 (3.07)	0.42 (0.40), 0.41	0.39 (1.03), 0.75	1.50 (0.72), 0.29
Potassium ^1^	28.47 (1.15)	23.70 (3.82)	25.34 (4.51)	39.00 (5.22)	1.45 (2.14), 0.57	0.48 (5.04), 0.93	7.48 (3.42), 0.27
**Coffee and Tea**							
	**Q1 ^2^** ***n* = 34,438,767** **% <EAR (SE)**	**Q2** ***n* = 2,439,053** **% <EAR (SE)**	**Q3** ***n* = 2,454,977** **% <EAR (SE)**	**Q4** ***n* = 2,434,713** **% <EAR (SE)**	**Quantile Trend ^3^** **Beta (SE),** ***p***	**Q1 vs. Q2, 3, 4 ^4^** **Beta (SE),** ***p***	**Q2–Q4 Trend ^5^** **Beta (SE),** ***p***
Calcium	62.91 (1.25)	77.82 (2.70)	68.57 (4.17)	63.34 (4.69)	1.96 (3.00), 0.58	7.48 (4.60), 0.25	−7.32 (1.19), 0.10
Magnesium	58.11 (0.93)	61.89 (2.90)	62.38 (2.62)	64.19 (3.42)	2.18 (0.36), 0.03	4.62 (0.74), 0.02	1.13 (0.39), 0.21
Phosphorus	24.55 (1.12)	31.86 (2.94)	26.06 (3.32)	22.22 (4.67)	0.24 (1.65), 0.90	2.49 (3.03), 0.50	−4.85 (0.58), 0.08
Vitamin A	38.13 (1.26)	44.44 (4.24)	44.11 (4.49)	50.64 (4.41)	3.94 (0.67), 0.03	7.99 (2.23), 0.07	2.97 (2.03), 0.38
Vitamin C	28.91 (1.45)	37.22 (4.51)	38.41 (5.87)	49.55 (5.77)	6.31 (0.87), 0.02 ^§^	12.30 (4.13), 0.10	5.97 (2.95), 0.29
Vitamin D	94.26 (0.50)	97.91 (0.79)	97.62 (0.91)	96.09 (1.85)	1.15 (0.66), 0.23	3.03 (0.59), 0.04	−0.88 (0.37), 0.25
Choline ^1^	3.88 (0.46)	3.48 (1.38)	4.69 (2.02)	5.42 (1.92)	0.42 (0.18), 0.14	0.61 (0.62), 0.42	0.97 (0.14), 0.09
Potassium ^1^	28.20 (1.18)	26.19 (3.23)	31.09 (3.59)	38.20 (4.62)	2.39 (1.17), 0.18	3.18 (3.72), 0.48	5.96 (0.65), 0.07
**Flavored Milk**							
	**Q1 ^2^** ***n* = 32,403,033** **% <EAR (SE)**	**Q2** ***n* = 2,768,510** **% <EAR (SE)**	**Q3** ***n* = 3,413,986** **% <EAR (SE)**	**Q4** ***n* = 3,181,981** **% <EAR (SE)**	**Quantile Trend ^3^** **Beta (SE),** ***p***	**Q1 vs. Q2, 3, 4 ^4^** **Beta (SE),** ***p***	**Q2–Q4 Trend ^5^** **Beta (SE),** ***p***
Calcium	69.34 (1.30)	59.57 (4.78)	48.09 (3.81)	19.93 (4.45)	−14.40 (2.28), 0.02 ^§^	−27.00 (13.11), 0.18	−19.97 (5.14), 0.16
Magnesium	63.59 (0.99)	46.12 (3.47)	42.70 (2.78)	37.14 (2.78)	−9.67 (1.63), 0.03 ^§^	−21.68 (2.89), 0.02 ^§^	−4.51 (0.66), 0.09
Phosphorus	29.62 (1.08)	15.89 (2.68)	9.52 (2.57)	1.11 (0.75)	−9.84 (0.78), 0.01 ^†^	−20.94 (4.73), 0.05 ^§^	−7.41 (0.62), 0.05
Vitamin A	45.13 (1.32)	21.93 (4.70)	20.71 (3.40)	13.23 (2.45)	−11.63 (2.33), 0.04 ^§^	−26.49 (3.06), 0.01 ^§^	−4.41 (1.93), 0.26
Vitamin C	32.18 (1.56)	23.72 (2.94)	27.23 (3.38)	30.50 (3.30)	−1.47 (1.52), 0.44	−4.93 (2.15), 0.15	3.39 (0.07), 0.01
Vitamin D	95.68 (0.43)	99.14 (0.68)	94.38 (2.03)	90.49 (3.86)	−1.18 (0.98), 0.35	−1.16 (2.75), 0.72	−4.32 (0.27), 0.04
Choline ^1^	3.58 (0.42)	2.01 (0.97)	5.37 (1.90)	6.24 (2.22)	0.78 (0.48), 0.24	1.02 (1.43), 0.55	2.11 (0.77), 0.22
Potassium ^1^	25.69 (1.17)	30.32 (3.64)	37.22 (3.25)	57.34 (3.98)	8.84 (1.90), 0.04 ^§^	16.02 (9.04), 0.22	13.63 (4.08), 0.19

EAR, estimated average requirement; AI, adequate intake^. 1^ >AI for choline and potassium. ^2^ Quantile 1 represents non-consumers; the remaining sample of those reporting intake (consumers) was divided into tertiles (quantiles 2, 3, and 4). ^3^ From regression analysis among all individuals, test for trend^. 4^ From regression analysis among all individuals, test for differences between non-consumers (Q1) and consumers (Q2, 3, 4). ^5^ From regression analysis among consumers (Q2–Q4), test for trend. ^†^ Statistically significant at *p* < 0.01. ^§^ Statistically significant at *p* < 0.05.

Analyses of 4 y cycle versus pooled sample: The results from the 4 y cycle analysis of added sugars intake from various beverages (soft drinks, fruit drinks, sport and energy drinks, and coffee and tea) and micronutrient adequacy among those 2–18 y are consistent with those from the pooled sample analysis (Supplemental Figure S2 and Table S5). While the statistical significance and effect sizes varied between the 4 y cycle and the pooled sample analyses, the directions of the associations were the same and their orders of magnitudes were similar.

### 3.3. Associations between Added Sugars from the Rest of the Diet and Micronutrient Adequacy

There were no significant associations between the added sugars from the rest of the diet and micronutrient adequacy among those aged 2–8 y; however, among those aged 9–18 y, significant associations emerged for all of the micronutrients examined (Supplemental Table S6). A higher added sugars intake was associated with significantly lower percentages of individuals with intakes below the EAR for calcium (a 14.3% unit decrease with each quartile increase, *p* = 0.02), magnesium (a 14.8% unit decrease, *p* = 0.01), phosphorus (a 14.3% unit decrease, *p* = 0.04), vitamin A (a 20.2% unit decrease, *p* = 0.03), vitamin C (a 7.6% unit decrease, *p* = 0.01), and vitamin D (a 3.1% unit decrease, *p* < 0.01), and significantly higher percentages with intakes above the AI for choline (a 1.7% unit increase with each quartile increase, *p* < 0.01) and potassium (a 12.9% unit increase, *p* = 0.02).

## 4. Discussion

Our examination of added sugars from beverages and micronutrient adequacy using the NHANES 2003–2018 data revealed some significant associations, which we deemed nutritionally significant in terms of the size of the difference in the percentage of individuals with a micronutrient intake below the EAR or above the AI. However, the associations varied by age and beverage type, with only one significant association among children compared to several among adolescents and teens. Among children (2–8 y), the association between added sugars from flavored milk and calcium adequacy followed a stepwise pattern, with percentages below the EAR for calcium being lower with every change to a higher added sugars quantile (increased intake) of flavored milk. Among adolescents and teens (9–18 y), a similar pattern for flavored milk and calcium adequacy was observed, and it also emerged for other micronutrients, with lower percentages below the EAR for magnesium, phosphorus, and vitamin A. Additionally, among those aged 9–18 y, higher added sugars from fruit drinks were associated with lower percentages of vitamin C intake below the EAR, with differences also following a stepwise pattern. In contrast, higher added sugars from either soft drinks or coffee and tea among those aged 9–18 y were associated with higher percentages below the EAR for magnesium and vitamins A and C, with the differences also being stepwise. For context, looking at the rest of the diet (excluding beverages with added sugars), higher added sugars intake among those aged 9–18 y was associated with lower percentages below the EAR or higher percentages above the AI for most of the micronutrients we examined.

The degree to which added sugars from beverages were associated with micronutrient adequacy was greater among adolescents and teens (9–18 y) versus children (2–8 y), likely driven by differences in beverage consumption and the overall diet. Those 9–18 y consumed more added sugars from soft drinks and coffee and tea compared to those 2–8 y, and it was only for the older age group that we observed differences in the nutritional significance of indicators of adequacy for magnesium and vitamins A and C that were associated with those beverages. In contrast, for added sugars from flavored milk, there were greater differences among those 2–8 y compared to those 9–18 y in the indicators of calcium adequacy, likely due to higher consumption of this beverage among children. Additionally, regarding the overall diet, there are more nutrients of concern due to low intakes in the diets of adolescents and teens compared to children [1], suggesting there is more room for improvement in the diets of adolescents and teens. For example, for added sugars from fruit drinks, there were differences in the indicators of vitamin C adequacy for both age groups, but these were only nutritionally significant among those 9–18 y for whom the percentages below the EAR changed from almost 50% to near zero at the highest level of added sugars intake. Likewise, for added sugars from flavored milk, the differences in the indicators of calcium adequacy were nutritionally significant for both age groups, but among those aged 9–18 y, there were also differences in the percentages below the EAR for magnesium, phosphorus, and vitamin A, and a percentage above the AI for potassium.

Considering the specific types of beverages with added sugars that we examined, the associations with micronutrient adequacy were, at times, in opposite directions: higher added sugars from soft drinks and coffee and tea were associated with higher percentages below the EAR, while higher added sugars from fruit drinks and flavored milk were associated with lower percentages below the EAR. Studies in other countries have found SSB consumption, defined in various ways, to be associated with lower intakes of several nutrients, such as calcium, magnesium, phosphorus, and vitamins A and D [11,12]; and in the US, soft drink consumption specifically, has been associated with lower intakes of calcium, magnesium, phosphorus, vitamin D, and potassium [7]. Similarly, among those aged 9–18 y, we observed higher added sugars from soft drinks and coffee and tea to be associated with higher percentages below the EAR for magnesium and vitamins A and C, and a lower percentage above the AI for potassium, suggesting these beverages may be displacing more nutrient-dense choices for this age group. In contrast, for both age groups, but more so among those aged 9–18 y, higher added sugars from fruit drinks were associated with a lower percentage below the EAR for vitamin C, and higher added sugars from flavored milk were associated with lower percentages below the EAR for calcium, magnesium, phosphorus, vitamin A, and potassium (higher percentages above the AI). These latter associations suggest that the vitamin C from fruit drinks can contribute to achieving vitamin C adequacy, and the nutrients provided by flavored milk, such as calcium, magnesium, phosphorus, vitamin A, and potassium, can contribute to achieving adequacy in these nutrients.

In addition to the potential direct effects of the specific beverages with added sugars that we examined, either in displacing more nutrient-dense beverage choices or contributing essential nutrients to the diet, their consumption may also be associated with other dietary components that can impact micronutrient adequacy. Other studies using NHANES data have demonstrated that SSB intake (as a whole) is associated with lower diet quality, as indicated by lower total scores on the HEI [8,9,10], and lower component scores for vegetables, total fruit, whole fruit, greens and beans, whole grains, dairy, seafood, plant proteins, and empty calories [10]; therefore, some of what we observed could be partly explained by this clustering of dietary choices. However, because the stepwise differences we observed in micronutrient adequacy corresponded to the specific beverages we examined, and hence, their nutrient composition (such as vitamin C in fruit drinks, and magnesium, phosphorus, and vitamin A in flavored milk), our findings suggest that some sources of added sugars can make important contributions to micronutrient intakes, which is consistent with other studies [6,12,13,14]. When we considered the overall diet, higher added sugars from the rest of the diet (excluding beverages with added sugars) among those 9–18 y were associated with lower percentages below the EAR (or higher percentages above the AI) for most micronutrients, likely reflecting the nutrient contribution from a variety of sources of added sugars. However, our findings for the rest of the diet are in contrast to those from a Canadian study, in which higher added sugars from solid food sources (excluding SSBs and flavored milk) were associated with lower intakes of several nutrients [11]; these differences might be due to different fortification practices between the US and Canada.

One of the strengths of our study was the pooling of data from eight NHANES cycles (2003–2018), providing the statistical power to examine beverages with added sugars at the granular level. Most studies on added sugars intake have examined SSBs as a group (grouping beverages with varying nutrient composition), which might dilute or mask important dietary associations. By examining specific beverages with different micronutrient compositions, our study makes a unique contribution to the literature on added sugars intake. Furthermore, we were able to stratify by age, and thus, examine associations among children (2–8 y) separately from associations among adolescents and teens (9–18 y), two age groups who differ considerably in their dietary behaviors. Another strength of our study was the use of micronutrient adequacy as the outcome, measured by the percentage of individuals with intakes below the EAR (or above the AI), which enabled us to identify differences that were nutritionally significant.

Like all analyses of dietary intake, ours was limited by the use of self-reported data, which are subject to errors of misreporting. The use of proxies to collect dietary intake data for young children (age 2–5 y) can also lead to underestimates of dietary intake [24]. Our added sugars quantiles were constructed from a pooled sample representing dietary intakes across a 16 y time span, and thus, our results may be confounded by changes over time in beverage and added sugars consumption. However, we observed similar trends in added sugars from beverages and micronutrient adequacy in our analyses of 4 y cycle samples. Lastly, due to the cross-sectional nature of NHANES data, we cannot infer causality between added sugars from beverages and micronutrient adequacy.

## 5. Conclusions

In conclusion, the patterns we observed suggest changes between 2003 and 2018 in the number of individuals consuming beverages with added sugars, and also in the volumes consumed and/or the added sugars content of the beverages themselves, with decreases for soft drinks and fruit drinks and increases for coffee and tea. For many of the nutrients of public health concern, we found nutritionally significant associations between the added sugars from beverages and micronutrient adequacy, and these were mainly among adolescents and teens (versus children). More specifically, higher added sugars from soft drinks and coffee and tea were associated with higher percentages below the EAR for magnesium and vitamins A and C, while higher added sugars from fruit drinks and flavored milk were associated with lower percentages below the EAR for calcium, magnesium, phosphorus, vitamin A, and potassium (higher percentage above the AI). As for the rest of the diet, higher added sugars intake among adolescents and teens was associated with lower percentages below the EAR for most micronutrients. Taken together, our results suggest the relationship between added sugars intake and micronutrient adequacy depends on the added sugar food and beverage sources and their nutrient composition. Given the changes over time in beverage consumption patterns, more research is needed to further characterize these changes; for example, to differentiate between changes in consumer behavior versus product formulations. Additionally, given the rapid pace of food product development, it will also be important to continue monitoring beverage consumption and added sugars intake trends and associations with micronutrient adequacy and diet quality.

## Figures and Tables

**Table 1:** Description of added sugar sources using WWEIA food categories.

Added Sugars Source	Description
Sweetened Beverages	Soft drinks; fruit drinks; sport and energy drinks; nutritional beverages; smoothies; grain drinks
Soft Drinks	
Fruit Drinks ^1^	
Sport and Energy Drinks	
Coffee and Tea	Coffee; tea
Flavored Milk	Flavored milk: whole, reduced fat, low-fat, non-fat
Rest of the Diet *	Milk and dairy (flavored milk excluded); protein foods; mixed dishes; grains; snacks and sweets; fruit; vegetables; beverages (sweetened beverages and coffee and tea excluded); alcoholic beverages; water, fats and oils; condiments and sauces; sugars; baby foods and formulas; other

^1^ Does not include 100% fruit juice. * All foods and beverages except sweetened beverages, coffee and tea, and flavored milk. WWEIA, What We Eat in America [20].

**Table 2:** Percentages of population reporting intake (consumers) of beverage sources of added sugars among children, adolescents, and teens from the pooled sample (NHANES 2003–2018, *n* = 21,005).

	Consumers (%) Mean ± SE	
Added Sugars Source	2–8 y (*n* = 8599)	9–18 y (*n* = 12,406)
Sweetened Beverages	68.55 ± 0.99	79.13 ± 0.66
Soft Drinks	36.20 ± 1.09	58.22 ± 0.83
Fruit Drinks	48.29 ± 0.93	39.56 ± 0.79
Sport and Energy Drinks	6.69 ± 0.54	12.25 ± 0.57
Coffee and Tea	9.86 ± 0.74	17.55 ± 0.76
Flavored Milk	32.64 ± 0.89	22.42 ± 0.85

**Table 3:** Added sugars intake (% kcal) from beverage sources and the rest of the diet among children, adolescents, and teens from the pooled sample (NHANES 2003–2018, *n* = 21,005).

Added Sugars Source	Added Sugars (% kcal) Mean ± SE			
	2–8 y (*n* = 8599)		9–18 y (*n* = 12,406)	
	All Individuals	Consumers Only	All Individuals	Consumers Only
Sweetened Beverages	3.72 ± 0.09	5.43 ± 0.10	6.61 ± 0.13	8.36 ± 0.14
Soft Drinks	1.44 ± 0.06	3.97 ± 0.11	4.26 ± 0.11	7.32 ± 0.14
Fruit Drinks	2.08 ± 0.06	4.31 ± 0.09	1.82 ± 0.05	4.59 ± 0.10
Sport and Energy Drinks	0.18 ± 0.02	2.75 ± 0.19	0.51 ± 0.04	4.13 ± 0.23
Coffee and Tea	0.29 ± 0.03	2.94 ± 0.18	0.80 ± 0.05	4.55 ± 0.25
Flavored Milk	0.81 ± 0.04	2.48 ± 0.07	0.43 ± 0.02	1.91 ± 0.05
Rest of the Diet *	9.01 ± 0.10	9.02 ± 0.10	8.23 ± 0.08	8.23 ± 0.08
Total Added Sugars	13.8 ± 0.12		16.1 ± 0.13	

* Given that almost the entire population consumes added sugars from the rest of the diet, the mean added sugars values for All Individuals and Consumers Only were almost identical within each age group.

**Table 4:** Quantiles of added sugars intake (% kcal) by beverage source among children, adolescents, and teens from the pooled sample (NHANES 2003–2018, *n* = 21,005).

Added Sugars Source	Quantile	2–8 y (*n* = 8599) Range (% kcal)	9–18 y (*n* = 12,406) Range (% kcal)
Sweetened Beverages	1 *	0	0
	2	>0 to ≤2.75	>0 to ≤4.67
	3	>2.75 to ≤6.16	>4.67 to ≤9.49
	4	>6.16	>9.49
Soft Drinks	1 *	0	0
	2	>0 to ≤2.16	>0 to ≤4.02
	3	>2.16 to ≤4.26	>4.02 to ≤7.89
	4	>4.26	>7.89
Fruit Drinks	1 *	0	0
	2	>0 to ≤2.18	>0 to ≤2.32
	3	>2.18 to ≤4.83	>2.32 to ≤4.90
	4	>4.83	>4.90
Sport and Energy Drinks	1 *	0	0
	2	>0 to ≤1.30	>0 to ≤2.26
	3	>1.30 to ≤2.70	>2.26 to ≤4.28
	4	>2.70	>4.28
Coffee and Tea	1 *	0	0
	2	>0 to ≤1.66	>0 to ≤2.22
	3	>1.66 to ≤2.81	>2.22 to ≤4.57
	4	>2.81	>4.57
Flavored Milk	1 *	0	0
	2	>0 to ≤1.42	>0 to ≤1.14
	3	>1.42 to ≤2.49	>1.14 to ≤2.03
	4	>2.49	>2.03

* Quantile 1 represents non-consumers; the remaining sample of those reporting intake (consumers) was divided into tertiles (quantiles 2, 3, and 4).

**Table 5:** Trends in % <EAR (>AI ^1^) for selected micronutrients by beverage source of added sugars among children (2–8 y, *n* = 8599) from the pooled sample (NHANES 2003–2018, *n* = 21,005); weighted n’s are shown in the table.

Sweetened Beverages							
	Q1 ^2^ *n* = 8,786,835 % <EAR (SE)	Q2 *n* = 6,338,167 % <EAR (SE)	Q3 *n* = 6,420,388 % <EAR (SE)	Q4 *n* = 6,392,984 % <EAR (SE)	Quantile Trend ^3^ Beta (SE), *p*	Q1 vs. Q2, 3, 4 ^4^ Beta (SE), *p*	Q2–Q4 Trend ^5^ Beta (SE), *p*
Calcium	15.72 (1.34)	15.78 (1.19)	25.41 (1.62)	24.68 (1.70)	3.59 (1.35), 0.12	6.48 (5.75), 0.38	4.33 (3.03), 0.39
Magnesium	0.64 (0.25)	0.58 (0.15)	0.80 (0.25)	0.74 (0.20)	0.05 (0.04), 0.33	0.07 (0.12), 0.64	0.08 (0.08), 0.52
Phosphorus	0.13 (0.07)	0.08 (0.04)	0.09 (0.04)	0.02 (0.02)	−0.03 (0.01), 0.08	−0.07 (0.04), 0.22	−0.03 (0.02), 0.42
Vitamin A	2.72 (0.63)	1.71 (0.50)	3.71 (0.67)	3.97 (0.75)	0.55 (0.37), 0.28	0.47 (1.33), 0.76	1.11 (0.51), 0.27
Vitamin C	1.78 (0.52)	0.99 (0.30)	0.46 (0.21)	0.79 (0.32)	−0.36 (0.18), 0.19	−1.04 (0.29), 0.07	−0.09 (0.25), 0.79
Vitamin D	85.50 (1.43)	87.00 (1.28)	90.42 (1.20)	90.43 (1.15)	1.82 (0.43), 0.05	3.87 (2.12), 0.21	1.67 (1.00), 0.34
Choline ^1^	47.09 (1.94)	45.26 (2.08)	41.38 (2.03)	42.02 (2.27)	−1.94 (0.59), 0.08	−4.25 (2.20), 0.19	−1.60 (1.33), 0.44
Potassium ^1^	42.22 (1.96)	41.65 (1.98)	38.28 (1.78)	39.55 (1.78)	−1.13 (0.58), 0.19	−2.46 (1.83), 0.31	−1.00 (1.36), 0.60
**Soft Drinks**							
	**Q1 ^2^** ***n* = 17,824,063** **% <EAR (SE)**	**Q2** ***n* = 3,322,242** **% <EAR (SE)**	**Q3** ***n* = 3,412,532** **% <EAR (SE)**	**Q4** ***n* = 3,379,537** **% <EAR (SE)**	**Quantile Trend ^3^** **Beta (SE), *p***	**Q1 vs. Q2, 3, 4 ^4^** **Beta (SE), *p***	**Q2–Q4 Trend ^5^** **Beta (SE), *p***
Calcium	17.48 (0.95)	22.54 (2.10)	24.05 (2.38)	27.04 (2.32)	3.27 (0.42), 0.02	6.95 (1.67), 0.05	2.23 (0.43), 0.12
Magnesium	0.61 (0.15)	0.92 (0.35)	0.55 (0.24)	0.89 (0.28)	0.07 (0.08), 0.46	0.18 (0.15), 0.35	−0.02 (0.20), 0.93
Phosphorus	0.12 (0.04)	0.11 (0.06)	0.05 (0.04)	0.02 (0.02)	−0.03 (0.01), 0.02	−0.06 (0.03), 0.23	−0.05 (0.01), 0.12
Vitamin A	2.46 (0.39)	3.04 (0.91)	3.31 (0.86)	5.14 (1.22)	0.76 (0.18), 0.05	1.32 (0.83), 0.25	1.03 (0.44), 0.26
Vitamin C	0.89 (0.23)	0.39 (0.21)	1.44 (0.55)	1.70 (0.83)	0.25 (0.17), 0.29	0.26 (0.52), 0.67	0.67 (0.22), 0.21
Vitamin D	87.54 (0.94)	90.02 (1.47)	88.80 (1.83)	89.89 (1.45)	0.79 (0.40), 0.19	2.04 (0.49), 0.05	−0.09 (0.66), 0.91
Choline ^1^	44.86 (1.40)	42.67 (2.57)	41.49 (3.11)	45.46 (3.01)	−0.43 (0.81), 0.65	−1.76 (1.41), 0.34	1.29 (1.47), 0.54
Potassium ^1^	41.83 (1.28)	38.80 (2.22)	38.03 (3.07)	38.65 (2.52)	−1.35 (0.49), 0.11	−3.32 (0.30), 0.01 ^†^	−0.09 (0.40), 0.85
**Fruit Drinks**							
	**Q1 ^2^** ***n* = 14,447,308** **% <EAR (SE)**	**Q2** ***n* = 4,473,337** **% <EAR (SE)**	**Q3** ***n* = 4,519,454** **% <EAR (SE)**	**Q4** ***n* = 4,498,275** **% <EAR (SE)**	**Quantile Trend ^3^** **Beta (SE), *p***	**Q1 vs. Q2, 3, 4 ^4^** **Beta (SE), *p***	**Q2–Q4 Trend ^5^** **Beta (SE), *p***
Calcium	17.84 (1.06)	17.50 (1.85)	25.52 (1.94)	23.33 (1.87)	2.31 (1.06), 0.16	4.45 (3.41), 0.32	2.76 (2.96), 0.52
Magnesium	0.82 (0.17)	0.48 (0.17)	0.51 (0.22)	0.38 (0.15)	−0.15 (0.04), 0.07	−0.37 (0.06), 0.02	−0.05 (0.04), 0.45
Phosphorus	0.09 (0.04)	0.06 (0.04)	0.06 (0.04)	0.01 (0.01)	−0.02 (0.00), 0.04	−0.05 (0.03), 0.19	−0.03 (0.01), 0.30
Vitamin A	2.87 (0.41)	2.31 (0.81)	2.44 (0.81)	3.17 (0.77)	0.03 (0.18), 0.87	−0.21 (0.40), 0.65	0.44 (0.17), 0.24
Vitamin C	1.79 (0.35)	0.16 (0.13)	0.03 (0.06)	0.02 (0.06)	−0.64 (0.24), 0.11	−1.72 (0.07), 0.00 ^†^	−0.07 (0.04), 0.32
Vitamin D	85.74 (0.98)	90.05 (1.26)	91.83 (1.28)	91.39 (1.31)	2.12 (0.65), 0.08	5.39 (0.76), 0.02 ^§^	0.63 (0.64), 0.50
Choline ^1^	47.01 (1.30)	42.52 (2.64)	40.30 (2.69)	38.69 (2.99)	−2.90 (0.41), 0.02	−6.58 (1.59), 0.05	−1.91 (0.18), 0.06
Potassium ^1^	42.11 (1.35)	37.00 (2.41)	37.41 (2.12)	41.23 (2.11)	−0.71 (1.23), 0.62	−3.44 (2.00), 0.23	2.16 (0.99), 0.27
**Sport and Energy Drinks**							
	**Q1 ^2^** ***n* = 26,070,488** **% <EAR (SE)**	**Q2** ***n* = 661,024** **% <EAR (SE)**	**Q3** ***n* = 642,393** **% <EAR (SE)**	**Q4** ***n* = 647,097** **% <EAR (SE)**	**Quantile Trend ^3^** **Beta (SE), *p***	**Q1 vs. Q2, 3, 4 ^4^** **Beta (SE), *p***	**Q2–Q4 Trend ^5^** **Beta (SE), *p***
Calcium	19.71 (0.81)	24.88 (4.89)	13.40 (3.96)	24.32 (4.15)	0.31 (1.80), 0.88	0.74 (3.94), 0.87	−0.04 (6.81), 1.00
Magnesium	0.58 (0.11)	1.72 (0.90)	1.01 (0.70)	2.94 (1.32)	0.63 (0.21), 0.10	1.28 (0.59), 0.16	0.64 (0.80), 0.57
Phosphorus	0.06 (0.02)	0.17 (0.20)	0.03 (0.09)	0.64 (0.42)	0.13 (0.07), 0.21	0.22 (0.19), 0.37	0.24 (0.23), 0.48
Vitamin A	2.92 (0.36)	4.71 (2.01)	1.55 (1.36)	2.48 (1.77)	−0.20 (0.41), 0.67	−0.12 (0.95), 0.91	−1.07 (1.24), 0.55
Vitamin C	1.11 (0.20)	0.04 (0.11)	2.52 (1.71)	0.02 (0.23)	−0.08 (0.40), 0.86	−0.16 (0.88), 0.87	−0.06 (1.52), 0.97
Vitamin D	88.17 (0.76)	90.21 (3.80)	83.59 (3.76)	90.57 (3.83)	−0.07 (1.09), 0.95	−0.28 (2.40), 0.92	0.33 (4.13), 0.95
Choline ^1^	44.55 (1.14)	42.09 (6.95)	44.84 (5.77)	39.60 (6.86)	−1.15 (0.64), 0.22	−2.28 (1.61), 0.29	−1.33 (2.43), 0.68
Potassium ^1^	40.19 (1.09)	38.63 (5.72)	46.08 (6.02)	38.34 (4.94)	0.42 (1.24), 0.77	1.09 (2.68), 0.72	−0.31 (4.62), 0.96
**Coffee and Tea**							
	**Q1 ^2^** ***n* = 25,183,581** **% <EAR (SE)**	**Q2** ***n* = 878,443** **% <EAR (SE)**	**Q3** ***n* = 951,216** **% <EAR (SE)**	**Q4** ***n* = 925,133** **% <EAR (SE)**	**Quantile Trend ^3^** **Beta (SE), *p***	**Q1 vs. Q2, 3, 4 ^4^** **Beta (SE), *p***	**Q2–Q4 Trend ^5^** **Beta (SE), *p***
Calcium	19.48 (0.83)	23.31 (4.90)	23.92 (4.37)	26.41 (5.56)	2.41 (0.33), 0.02	4.88 (0.96), 0.04	1.49 (0.54), 0.22
Magnesium	0.64 (0.11)	0.61 (0.52)	0.83 (0.48)	0.87 (0.80)	0.07 (0.03), 0.11	0.11 (0.09), 0.35	0.14 (0.05), 0.22
Phosphorus	0.07 (0.02)	0.11 (0.11)	0.01 (0.09)	0.17 (0.25)	0.01 (0.02), 0.56	0.03 (0.05), 0.61	0.02 (0.07), 0.85
Vitamin A	2.68 (0.33)	4.84 (2.78)	3.95 (1.50)	6.33 (3.39)	1.12 (0.31), 0.07	2.28 (0.69), 0.08	0.64 (0.94), 0.62
Vitamin C	0.89 (0.18)	3.57 (1.77)	0.36 (0.60)	2.46 (1.85)	0.46 (0.59), 0.51	1.32 (1.02), 0.32	−0.72 (1.53), 0.72
Vitamin D	87.77 (0.78)	93.36 (2.10)	91.67 (2.71)	87.51 (3.01)	1.03 (1.26), 0.50	3.43 (1.77), 0.19	−2.85 (0.71), 0.16
Choline ^1^	44.65 (1.16)	41.82 (4.58)	45.58 (4.91)	39.65 (4.37)	−1.08 (0.83), 0.32	−2.14 (1.75), 0.35	−0.78 (2.83), 0.83
Potassium ^1^	40.29 (1.01)	38.68 (5.09)	37.62 (3.98)	45.99 (5.33)	0.60 (1.19), 0.66	0.06 (2.60), 0.98	3.36 (2.71), 0.43
**Flavored Milk**							
	**Q1 ^2^** ***n* = 18,819,476** **% <EAR (SE)**	**Q2** ***n* = 3,028,928** **% <EAR (SE)**	**Q3** ***n* = 2,928,578** **% <EAR (SE)**	**Q4** ***n* = 3,161,392** **% <EAR (SE)**	**Quantile Trend ^3^** **Beta (SE), *p***	**Q1 vs. Q2, 3, 4 ^4^** **Beta (SE), *p***	**Q2–Q4 Trend ^5^** **Beta (SE), *p***
Calcium	23.88 (0.94)	19.22 (3.26)	10.13 (2.16)	0.61 (0.67)	−7.39 (0.70), 0.01 ^†^	−13.39 (6.58), 0.18	−9.30 (0.12), 0.01 ^†^
Magnesium	1.00 (0.17)	0.39 (0.26)	0.11 (0.09)	0.00 (0.00)	−0.38 (0.07), 0.03	−0.82 (0.14), 0.03	−0.20 (0.05), 0.15
Phosphorus	0.14 (0.05)	0.00 (0.01)	0.00 (0.00)	0.00 (0.00)	−0.06 (0.02), 0.11	−0.14 (0.00), 0.00 ^†^	0.00 (0.00), 0.32
Vitamin A	4.27 (0.44)	0.53 (0.55)	0.31 (0.22)	0.12 (0.15)	−1.65 (0.52), 0.09	−3.94 (0.14), 0.00 ^†^	−0.20 (0.01), 0.02
Vitamin C	0.89 (0.22)	1.04 (0.59)	0.87 (0.44)	1.77 (0.64)	0.21 (0.11), 0.20	0.33 (0.33), 0.42	0.35 (0.30), 0.45
Vitamin D	89.56 (0.69)	98.38 (1.01)	92.49 (2.31)	79.09 (4.78)	−1.66 (2.95), 0.63	0.85 (6.92), 0.91	−9.56 (2.07), 0.14
Choline ^1^	43.11 (1.28)	32.67 (2.88)	44.00 (4.12)	64.39 (3.60)	4.50 (4.18), 0.39	2.90 (11.23), 0.82	15.71 (2.53), 0.10
Potassium ^1^	37.29 (1.05)	32.02 (2.54)	44.17 (3.62)	68.76 (3.52)	7.97 (3.89), 0.18	10.22 (13.13), 0.52	18.22 (3.44), 0.12

EAR, estimated average requirement; AI, adequate intake^. 1^ >AI for choline and potassium. ^2^ Quantile 1 represents non-consumers; the remaining sample of those reporting intake (consumers) was divided into tertiles (quantiles 2, 3, and 4)^. 3^ From regression analysis among all individuals, test for trend. ^4^ From regression analysis among all individuals, test for differences between non-consumers (Q1) and consumers (Q2, 3, 4). ^5^ From regression analysis among consumers (Q2–Q4), test for trend. ^†^ Statistically significant at *p* < 0.01. ^§^ Statistically significant at *p* < 0.05.

## Data Availability

The data used in this manuscript are publicly available at the NHANES website: https://wwwn.cdc.gov/nchs/nhanes/ (accessed on 20 June 2023).

## Supplementary Materials

The following supporting information can be downloaded at: https://www.mdpi.com/article/10.3390/nu15153285/s1, Figure S1. NHANES 2003–2018 analytic sample flow chart. Figure S2. Change in % <EAR (>AI ^†^) in association with increased added sugars intake from select beverage sources: (A) soft drinks and (B) fruit drinks, by NHANES 4-y cycles, among children, adolescents and teens (2–18 y). (C) sport and energy drinks and (D) coffee and tea, by NHANES 4-y cycles, among children, adolescents and teens (2–18 y). Table S1. Percent of children, adolescents and teens (2–18 y) reporting intake (consumers) of beverage sources of added sugars from the pooled sample (NHANES 2003-2018, *n* = 21,005). Table S2. Trends in added sugars intake (% kcal) from beverage sources and the rest of the diet among all children, adolescents and teens and consumers only from the pooled sample (NHANES 2003–2018, *n* = 21,005). Table S3. Trends in added sugars intake (grams) from beverage sources and the rest of the diet among all children, adolescents and teens and consumers only from the pooled sample (NHANES 2003–2018, *n* = 21,005). Table S4. Trends in % <EAR (>AI ^1^) for selected micronutrients by beverage source of added sugars and the rest of the diet among children, adolescents and teens (2–18 y) from the pooled sample (NHANES 2003-2018, *n* = 21,005); weighted n’s are shown in the table. Table S5. Association between added sugars intake from select beverages and % <EAR (>AI ^1^) among children, adolescents and teens (2–18 y) by NHANES 4-y cycles. Table S6. Quartiles of added sugars intake (% kcal) from the rest of the diet and trends in % <EAR (>AI ^1^) for selected micronutrients among children, adolescents and teens from the pooled sample (NHANES 2003-2018, *n* = 21,005); weighted n’s are shown in the table.

## Abbreviations

AI	Adequate intake
DGA	Dietary Guidelines for Americans
EAR	Estimated average requirement
FPED	Food Patterns Equivalent Database
HEI	Healthy Eating Index
NCI	National Cancer Institute
SSB	Sugar-sweetened beverages
UI	Usual intake
WWEIA	What We Eat in America
