# Supplementary material for: Intakes of Added Sugars, with a Focus on Beverages and the Associations with Micronutrient Adequacy in US Children, Adolescents, and Teens (NHANES 2003–2018)
Source: Nutrients. 2023 Jul 25;15(15):3285. doi: 10.3390/nu15153285 (PMC10420670; doi:10.3390/nu15153285)
Supplement: Supplementary file 1 [file nutrients-15-03285-s001.zip › nutrients-2503720-supplementary.pdf]

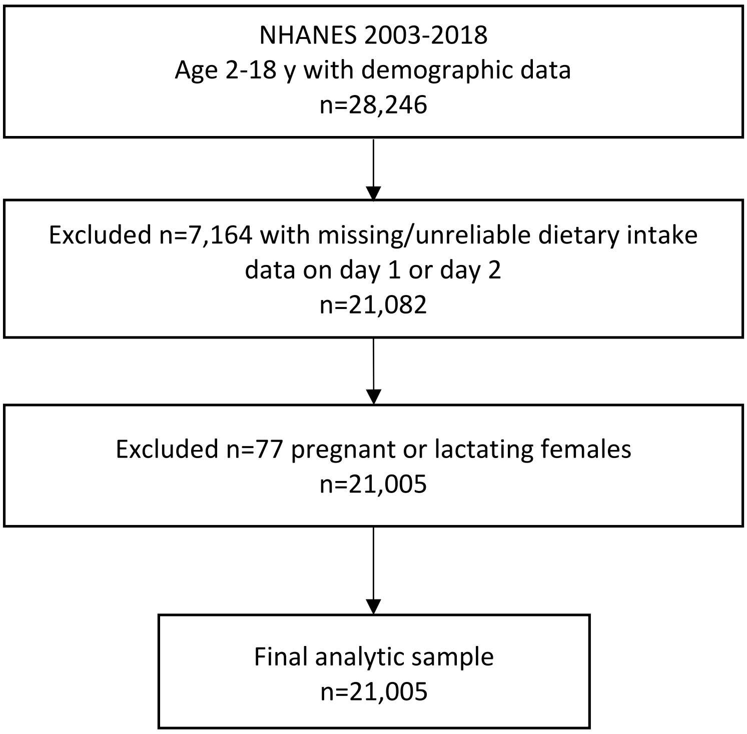

Supplemental Figure S1. NHANES 2003-2018 analytic sample flow chart.

Supplemental Table S1. Percent of children, adolescents and teens (2-18 y) reporting intake (consumers) of beverage sources of added sugars from the pooled sample (NHANES 2003-2018, n=21,005).

**Food Group**	**Sweetened Beverages**		**Soft Drinks**		**Fruit Drinks**		**Sport and Energy Drinks**		**Coffee and Tea**		**Flavored Milk**	
**2-8 y (n=8,599)**												
**Year**	**Mean ± SE**	**P**	**Mean ± SE**	**P**	**Mean ± SE**	**P**	**Mean ± SE**	**P**	**Mean ± SE**	**P**	**Mean ± SE**	**P**
**2003-04**	82.16 ± 1.82		55.23 ± 2.52		57.93 ± 2.62		0.12 ± 0.08		12.98 ± 3.06		35.27 ± 1.99	
**2005-06**	73.16 ± 2.02	<0.01*	42.04 ± 1.76	<0.01*	51.36 ± 2.79	0.09	8.83 ± 2.11	<0.01*	6.85 ± 1.30	0.07	30.81 ± 2.53	0.17
**2007-08**	75.21 ± 1.79	0.01	42.15 ± 2.89	<0.01*	54.00 ± 2.19	0.25	6.34 ± 2.04	<0.01*	12.16 ± 2.68	0.84	35.35 ± 2.75	0.98
**2009-10**	67.97 ± 1.93	<0.01*	33.69 ± 3.08	<0.01*	48.51 ± 1.94	<0.01*	6.04 ± 1.51	<0.01*	8.31 ± 1.23	0.16	36.18 ± 1.92	0.74
**2011-12**	71.28 ± 3.26	<0.01*	38.09 ± 4.17	<0.01*	50.60 ± 2.43	0.04	7.38 ± 1.31	<0.01*	10.18 ± 1.33	0.40	34.77 ± 3.56	0.90
**2013-14**	58.42 ± 3.26	<0.01*	25.45 ± 2.65	<0.01*	42.95 ± 3.39	<0.01*	9.12 ± 1.82	<0.01*	9.15 ± 1.93	0.29	29.77 ± 1.50	0.03
**2015-16**	59.59 ± 2.85	<0.01*	25.11 ± 2.50	<0.01*	43.02 ± 1.67	<0.01*	6.51 ± 0.92	<0.01*	8.39 ± 1.97	0.21	26.57 ± 1.83	<0.01*
**2017-18**	60.66 ± 2.97	<0.01*	28.17 ± 2.32	<0.01*	37.91 ± 3.15	<0.01*	9.12 ± 1.31	<0.01*	10.89 ± 2.28	0.58	32.29 ± 2.99	0.41
**Trend**[1]	**Beta ± SE**	**P**	**Beta ± SE**	**P**	**Beta ± SE**	**P**	**Beta ± SE**	**P**	**Beta ± SE**	**P**	**Beta ± SE**	**P**
	-3.15 ± 0.39	<0.01	-3.80 ± 0.38	<0.01	-2.53 ± 0.42	<0.01	0.72 ± 0.20	<0.01	-0.17 ± 0.37	0.65	-0.73 ± 0.37	0.05
**9-18 y (n=12,406)**												
**Year**	**Mean ± SE**	**P**	**Mean ± SE**	**P**	**Mean ± SE**	**P**	**Mean ± SE**	**P**	**Mean ± SE**	**P**	**Mean ± SE**	**P**
**2003-04**	89.91 ± 1.28		74.99 ± 2.34		50.94 ± 2.14		0.27 ± 0.13		16.39 ± 2.16		20.36 ± 1.58	
**2005-06**	83.99 ± 2.13	0.02	68.05 ± 2.26	0.03	40.08 ± 2.21	<0.01*	15.13 ± 1.69	<0.01*	13.27 ± 1.60	0.25	19.76 ± 1.84	0.81
**2007-08**	80.01 ± 1.94	<0.01*	60.50 ± 2.64	<0.01*	39.15 ± 1.95	<0.01*	14.21 ± 1.87	<0.01*	19.03 ± 2.62	0.44	22.78 ± 3.05	0.48
**2009-10**	78.71 ± 1.73	<0.01*	58.80 ± 2.21	<0.01*	40.23 ± 3.14	0.01	14.06 ± 2.13	<0.01*	15.77 ± 2.51	0.85	28.59 ± 2.72	0.01
**2011-12**	82.78 ± 2.24	0.01	57.16 ± 2.52	<0.01*	42.98 ± 2.05	0.01	15.60 ± 1.52	<0.01*	19.89 ± 1.95	0.23	22.33 ± 2.41	0.50
**2013-14**	73.95 ± 1.48	<0.01*	51.21 ± 1.60	<0.01*	37.46 ± 1.98	<0.01*	14.76 ± 1.59	<0.01*	18.66 ± 1.56	0.40	22.22 ± 1.97	0.46
**2015-16**	72.71 ± 2.11	<0.01*	50.61 ± 2.43	<0.01*	32.31 ± 1.64	<0.01*	11.43 ± 1.47	<0.01*	16.58 ± 2.58	0.96	23.46 ± 2.82	0.34
**2017-18**	71.57 ± 2.04	<0.01*	45.13 ± 2.39	<0.01*	33.88 ± 2.33	<0.01*	12.52 ± 1.20	<0.01*	20.72 ± 1.72	0.12	19.87 ± 2.35	0.86
**Trend**[1]	**Beta ± SE**	**P**	**Beta ± SE**	**P**	**Beta ± SE**	**P**	**Beta ± SE**	**P**	**Beta ± SE**	**P**	**Beta ± SE**	**P**
	-2.37 ± 0.28	<0.01	-3.87 ± 0.36	<0.01	-1.91 ± 0.32	<0.01	0.82 ± 0.20	<0.01	0.59 ± 0.31	0.06	0.08 ± 0.34	0.82
**2-18 y (n=21,005)**												
**Year**	**Mean ± SE**	**P**	**Mean ± SE**	**P**	**Mean ± SE**	**P**	**Mean ± SE**	**P**	**Mean ± SE**	**P**	**Mean ± SE**	**P**
**2003-04**	86.83 ± 0.95		67.13 ± 1.81		53.72 ± 1.67		0.21 ± 0.07		15.04 ± 1.67		26.29 ± 1.25	
**2005-06**	79.69 ± 1.55	<0.01*	57.73 ± 1.52	<0.01*	44.55 ± 1.98	<0.01*	12.63 ± 1.59	<0.01*	10.72 ± 0.97	0.03	24.14 ± 1.73	0.32
**2007-08**	78.10 ± 1.35	<0.01*	53.18 ± 2.04	<0.01*	45.07 ± 1.66	<0.01*	11.07 ± 1.24	<0.01*	16.29 ± 2.15	0.65	27.79 ± 2.41	0.58
**2009-10**	74.37 ± 1.71	<0.01*	48.67 ± 2.31	<0.01*	43.57 ± 2.31	<0.01*	10.82 ± 1.52	<0.01*	12.76 ± 1.90	0.37	31.65 ± 2.04	0.03
**2011-12**	77.93 ± 2.31	<0.01*	49.13 ± 2.88	<0.01*	46.19 ± 1.68	<0.01*	12.14 ± 1.24	<0.01*	15.80 ± 1.03	0.70	27.57 ± 2.63	0.66
**2013-14**	67.84 ± 1.55	<0.01*	41.07 ± 1.87	<0.01*	39.62 ± 1.86	<0.01*	12.54 ± 1.35	<0.01*	14.91 ± 1.41	0.96	25.20 ± 1.49	0.57
**2015-16**	67.42 ± 2.04	<0.01*	40.33 ± 2.34	<0.01*	36.63 ± 1.37	<0.01*	9.45 ± 0.80	<0.01*	13.28 ± 2.29	0.54	24.71 ± 2.07	0.52
**2017-18**	67.31 ± 2.10	<0.01*	38.50 ± 1.98	<0.01*	35.45 ± 2.04	<0.01*	11.19 ± 0.74	<0.01*	16.88 ± 1.85	0.46	24.73 ± 1.82	0.48
**Trend**[1]	**Beta ± SE**	**P**	**Beta ± SE**	**P**	**Beta ± SE**	**P**	**Beta ± SE**	**P**	**Beta ± SE**	**P**	**Beta ± SE**	**P**
	-2.68 ± 0.26	<0.01	-3.84 ± 0.30	<0.01	-2.16 ± 0.28	<0.01	0.78 ± 0.15	<0.01	0.29 ± 0.27	0.29	-0.24 ± 0.27	0.36

*Significantly different from reference cycle (2003-04), p<0.01

[1]Linear trend across all cycles, statistically significant at p<0.01

Supplemental Table S2. Trends in added sugars intake (% kcal) from beverage sources and the rest of the diet among all children, adolescents and teens and consumers only from the pooled sample (NHANES 2003-2018, n=21,005).

**Food Group**	**Sweetened Beverages**		**Soft Drinks**		**Fruit Drinks**		**Sport and Energy Drinks**		**Coffee and Tea**		**Flavored Milk**		**Rest of the Diet**	
**All Individuals 2-8 y**														
**Year**	**Mean ± SE**	**P**	**Mean ± SE**	**P**	**Mean ± SE**	**P**	**Mean ± SE**	**P**	**Mean ± SE**	**P**	**Mean ± SE**	**P**	**Mean ± SE**	**P**
**2003-04**	5.30 ± 0.26		2.55 ± 0.22		2.73 ± 0.18		0.004 ± 0.003		0.38 ± 0.13		0.84 ± 0.09		8.95 ± 0.26	
**2005-06**	4.23 ± 0.20	<0.01*	1.66 ± 0.13	<0.01*	2.24 ± 0.14	0.03	0.29 ± 0.10	0.01	0.14 ± 0.03	0.07	0.74 ± 0.08	0.36	9.78 ± 0.27	0.03
**2007-08**	4.51 ± 0.21	0.02	1.71 ± 0.19	<0.01*	2.67 ± 0.15	0.78	0.12 ± 0.03	<0.01*	0.29 ± 0.08	0.58	0.94 ± 0.11	0.50	9.27 ± 0.21	0.34
**2009-10**	3.68 ± 0.15	<0.01*	1.23 ± 0.12	<0.01*	2.27 ± 0.15	0.05	0.16 ± 0.05	<0.01*	0.28 ± 0.03	0.48	0.98 ± 0.08	0.27	8.53 ± 0.23	0.23
**2011-12**	3.73 ± 0.25	<0.01*	1.44 ± 0.22	<0.01*	2.10 ± 0.14	0.01	0.19 ± 0.05	<0.01*	0.28 ± 0.05	0.50	0.88 ± 0.16	0.85	9.02 ± 0.28	0.85
**2013-14**	2.97 ± 0.27	<0.01*	0.95 ± 0.10	<0.01*	1.71 ± 0.23	<0.01*	0.28 ± 0.08	<0.01*	0.34 ± 0.11	0.80	0.68 ± 0.06	0.12	9.26 ± 0.35	0.48
**2015-16**	2.82 ± 0.19	<0.01*	0.98 ± 0.11	<0.01*	1.62 ± 0.10	<0.01*	0.19 ± 0.05	<0.01*	0.23 ± 0.06	0.31	0.61 ± 0.06	0.03	8.57 ± 0.25	0.29
**2017-18**	2.58 ± 0.22	<0.01*	0.99 ± 0.17	<0.01*	1.31 ± 0.12	<0.01*	0.23 ± 0.05	<0.01*	0.38 ± 0.12	1.00	0.81 ± 0.11	0.78	8.79 ± 0.30	0.69
**Trend[1]**	**Beta ± SE**	**P**	**Beta ± SE**	**P**	**Beta ± SE**	**P**	**Beta ± SE**	**P**	**Beta ± SE**	**P**	**Beta ± SE**	**P**	**Beta ± SE**	**P**
	-0.37 ± 0.04	<0.01	-0.19 ± 0.03	<0.01	-0.19 ± 0.02	<0.01	0.02 ± 0.01	0.02	0.01 ± 0.02	0.66	-0.02 ± 0.01	0.13	-0.08 ± 0.04	0.06
**All Individuals 9-18 y**														
**Year**	**Mean ± SE**	**P**	**Mean ± SE**	**P**	**Mean ± SE**	**P**	**Mean ± SE**	**P**	**Mean ± SE**	**P**	**Mean ± SE**	**P**	**Mean ± SE**	**P**
**2003-04**	9.18 ± 0.43		6.54 ± 0.33		2.62 ± 0.23		0.01 ± 0.003		0.53 ± 0.08		0.39 ± 0.04		8.46 ± 0.21	
**2005-06**	7.93 ± 0.46	0.05	5.42 ± 0.33	0.02	1.86 ± 0.13	0.01	0.62 ± 0.08	<0.01*	0.38 ± 0.06	0.13	0.41 ± 0.04	0.77	8.48 ± 0.18	0.95
**2007-08**	7.56 ± 0.50	0.01	4.92 ± 0.51	0.01	2.04 ± 0.09	0.02	0.58 ± 0.09	<0.01*	0.67 ± 0.12	0.37	0.40 ± 0.05	0.93	8.65 ± 0.23	0.54
**2009-10**	6.67 ± 0.36	<0.01*	4.09 ± 0.21	<0.01*	2.05 ± 0.22	0.07	0.50 ± 0.10	<0.01*	0.64 ± 0.11	0.44	0.63 ± 0.08	0.01	8.37 ± 0.27	0.79
**2011-12**	6.38 ± 0.21	<0.01*	3.76 ± 0.21	<0.01*	1.98 ± 0.15	0.02	0.64 ± 0.07	<0.01*	1.23 ± 0.18	<0.01*	0.41 ± 0.05	0.72	7.83 ± 0.24	0.05
**2013-14**	5.86 ± 0.27	<0.01*	3.60 ± 0.23	<0.01*	1.43 ± 0.12	<0.01*	0.80 ± 0.21	<0.01*	1.14 ± 0.29	0.04	0.40 ± 0.05	0.96	7.78 ± 0.21	0.02
**2015-16**	4.78 ± 0.26	<0.01*	3.06 ± 0.20	<0.01*	1.26 ± 0.10	<0.01*	0.41 ± 0.07	<0.01*	0.86 ± 0.14	0.04	0.42 ± 0.06	0.67	7.83 ± 0.22	0.04
**2017-18**	4.67 ± 0.30	<0.01*	2.76 ± 0.28	<0.01*	1.33 ± 0.14	<0.01*	0.49 ± 0.05	<0.01*	0.94 ± 0.09	<0.01*	0.35 ± 0.04	0.42	8.40 ± 0.26	0.84
**Trend[1]**	**Beta ± SE**	**P**	**Beta ± SE**	**P**	**Beta ± SE**	**P**	**Beta ± SE**	**P**	**Beta ± SE**	**P**	**Beta ± SE**	**P**	**Beta ± SE**	**P**
	-0.63 ± 0.06	<0.01	-0.51 ± 0.05	<0.01	-0.17 ± 0.03	<0.01	0.04 ± 0.01	<0.01	0.09 ± 0.02	<0.01	-0.01 ± 0.01	0.42	-0.08 ± 0.03	0.02
**All Individuals 2-18 y**														
**Year**	**Mean ± SE**	**P**	**Mean ± SE**	**P**	**Mean ± SE**	**P**	**Mean ± SE**	**P**	**Mean ± SE**	**P**	**Mean ± SE**	**P**	**Mean ± SE**	**P**
**2003-04**	7.64 ± 0.31		4.95 ± 0.25		2.67 ± 0.15		0.01 ± 0.002		0.47 ± 0.07		0.57 ± 0.04		8.65 ± 0.13	
**2005-06**	6.46 ± 0.29	0.01	3.93 ± 0.21	<0.01*	2.01 ± 0.10	<0.01*	0.49 ± 0.08	<0.01*	0.29 ± 0.03	0.02	0.54 ± 0.05	0.61	9.00 ± 0.16	0.10

**2007-08**	6.34 ± 0.33	0.01	3.64 ± 0.34	<0.01*	2.29 ± 0.07	0.03	0.39 ± 0.06	<0.01*	0.52 ± 0.09	0.68	0.62 ± 0.06	0.58	8.90 ± 0.14	0.21
**2009-10**	5.46 ± 0.26	<0.01*	2.94 ± 0.16	<0.01*	2.14 ± 0.17	0.02	0.37 ± 0.07	<0.01*	0.50 ± 0.07	0.81	0.77 ± 0.05	<0.01*	8.43 ± 0.18	0.32
**2011-12**	5.27 ± 0.18	<0.01*	2.78 ± 0.19	<0.01*	2.03 ± 0.11	<0.01*	0.45 ± 0.05	<0.01*	0.83 ± 0.11	0.01	0.61 ± 0.09	0.70	8.33 ± 0.22	0.20
**2013-14**	4.72 ± 0.23	<0.01*	2.55 ± 0.18	<0.01*	1.54 ± 0.12	<0.01*	0.60 ± 0.14	<0.01*	0.82 ± 0.20	0.10	0.51 ± 0.04	0.28	8.36 ± 0.25	0.30
**2015-16**	3.99 ± 0.20	<0.01*	2.22 ± 0.15	<0.01*	1.40 ± 0.07	<0.01*	0.32 ± 0.06	<0.01*	0.61 ± 0.10	0.28	0.50 ± 0.05	0.24	8.13 ± 0.20	0.03
**2017-18**	3.85 ± 0.21	<0.01*	2.07 ± 0.21	<0.01*	1.32 ± 0.11	<0.01*	0.39 ± 0.03	<0.01*	0.72 ± 0.10	0.04	0.53 ± 0.06	0.53	8.55 ± 0.22	0.68
**Trend[1]**	**Beta ± SE**	**P**	**Beta ± SE**	**P**	**Beta ± SE**	**P**	**Beta ± SE**	**P**	**Beta ± SE**	**P**	**Beta ± SE**	**P**	**Beta ± SE**	**P**
	-0.52 ± 0.04	<0.01	-0.38 ± 0.03	<0.01	-0.18 ± 0.02	<0.01	0.03 ± 0.01	<0.01	0.05 ± 0.01	<0.01	-0.01 ± 0.01	0.12	-0.08 ± 0.03	0.01
**Consumers Only 2-8 y**														
**Year**	**Mean ± SE**	**P**	**Mean ± SE**	**P**	**Mean ± SE**	**P**	**Mean ± SE**	**P**	**Mean ± SE**	**P**	**Mean ± SE**	**P**	**Mean ± SE**	**P**
**2003-04**	6.45 ± 0.33		4.61 ± 0.27		4.72 ± 0.28		3.79 ± 0.40		2.90 ± 0.65		2.40 ± 0.19		8.95 ± 0.26	
**2005-06**	5.79 ± 0.21	0.10	3.94 ± 0.23	0.06	4.37 ± 0.19	0.30	3.29 ± 0.55	0.47	2.01 ± 0.26	0.21	2.39 ± 0.15	0.98	9.78 ± 0.27	0.03
**2007-08**	6.00 ± 0.20	0.25	4.05 ± 0.30	0.17	4.94 ± 0.25	0.56	1.86 ± 0.35	<0.01*	2.41 ± 0.30	0.50	2.66 ± 0.20	0.35	9.28 ± 0.21	0.33
**2009-10**	5.42 ± 0.18	0.01	3.66 ± 0.21	0.01	4.68 ± 0.18	0.90	2.67 ± 0.36	0.04	3.42 ± 0.29	0.47	2.70 ± 0.16	0.23	8.53 ± 0.23	0.24
**2011-12**	5.24 ± 0.35	0.01	3.79 ± 0.40	0.09	4.14 ± 0.27	0.14	2.53 ± 0.36	0.02	2.80 ± 0.39	0.90	2.53 ± 0.26	0.68	9.02 ± 0.28	0.85
**2013-14**	5.08 ± 0.28	<0.01*	3.72 ± 0.26	0.02	3.97 ± 0.26	0.05	3.12 ± 0.57	0.34	3.66 ± 0.67	0.42	2.29 ± 0.17	0.70	9.26 ± 0.35	0.48
**2015-16**	4.72 ± 0.15	<0.01*	3.91 ± 0.25	0.06	3.75 ± 0.17	<0.01*	2.96 ± 0.72	0.32	2.78 ± 0.28	0.87	2.31 ± 0.22	0.76	8.57 ± 0.25	0.30
**2017-18**	4.25 ± 0.29	<0.01*	3.52 ± 0.40	0.03	3.45 ± 0.20	<0.01*	2.55 ± 0.29	0.01	3.45 ± 0.51	0.51	2.49 ± 0.18	0.71	8.79 ± 0.30	0.69
**Trend[1]**	**Beta ± SE**	**P**	**Beta ± SE**	**P**	**Beta ± SE**	**P**	**Beta ± SE**	**P**	**Beta ± SE**	**P**	**Beta ± SE**	**P**	**Beta ± SE**	**P**
	-0.28 ± 0.04	<0.01	-0.12 ± 0.05	0.02	-0.18 ± 0.04	<0.01	-0.01 ± 0.10	0.93	0.11 ± 0.09	0.20	-0.01 ± 0.03	0.72	-0.08 ± 0.04	0.06
**Consumers Only 9-18 y**														
**Year**	**Mean ± SE**	**P**	**Mean ± SE**	**P**	**Mean ± SE**	**P**	**Mean ± SE**	**P**	**Mean ± SE**	**P**	**Mean ± SE**	**P**	**Mean ± SE**	**P**
**2003-04**	10.21 ± 0.42		8.73 ± 0.33		5.15 ± 0.33		2.13 ± 0.69		3.26 ± 0.24		1.93 ± 0.08		8.46 ± 0.21	
**2005-06**	9.44 ± 0.40	0.18	7.97 ± 0.34	0.11	4.65 ± 0.16	0.18	4.12 ± 0.22	0.01	2.90 ± 0.14	0.19	2.07 ± 0.16	0.43	8.48 ± 0.18	0.95
**2007-08**	9.45 ± 0.51	0.25	8.13 ± 0.63	0.40	5.20 ± 0.17	0.88	4.06 ± 0.42	0.02	3.51 ± 0.33	0.54	1.75 ± 0.11	0.20	8.65 ± 0.23	0.54
**2009-10**	8.47 ± 0.42	<0.01*	6.96 ± 0.29	<0.01*	5.09 ± 0.29	0.90	3.59 ± 0.29	0.06	4.05 ± 0.31	0.05	2.22 ± 0.14	0.08	8.38 ± 0.27	0.80
**2011-12**	7.71 ± 0.27	<0.01*	6.58 ± 0.26	<0.01*	4.60 ± 0.35	0.25	4.08 ± 0.27	0.01	6.20 ± 0.58	<0.01*	1.86 ± 0.09	0.53	7.83 ± 0.24	0.05
**2013-14**	7.92 ± 0.35	<0.01*	7.02 ± 0.36	<0.01*	3.82 ± 0.18	<0.01*	5.42 ± 1.22	0.02	6.13 ± 1.70	0.10	1.79 ± 0.15	0.39	7.79 ± 0.22	0.02
**2015-16**	6.57 ± 0.28	<0.01*	6.04 ± 0.21	<0.01*	3.90 ± 0.25	<0.01*	3.59 ± 0.63	0.12	5.17 ± 0.34	<0.01*	1.81 ± 0.12	0.38	7.86 ± 0.23	0.05
**2017-18**	6.52 ± 0.38	<0.01*	6.13 ± 0.43	<0.01*	3.94 ± 0.32	0.01	3.89 ± 0.41	0.03	4.53 ± 0.37	<0.01*	1.77 ± 0.19	0.41	8.42 ± 0.26	0.90
**Trend[1]**	**Beta ± SE**	**P**	**Beta ± SE**	**P**	**Beta ± SE**	**P**	**Beta ± SE**	**P**	**Beta ± SE**	**P**	**Beta ± SE**	**P**	**Beta ± SE**	**P**
	-0.54 ± 0.06	<0.01	-0.38 ± 0.06	<0.01	-0.20 ± 0.05	<0.01	0.02 ± 0.09	0.79	0.33 ± 0.07	<0.01	-0.03 ± 0.02	0.13	-0.08 ± 0.03	0.03
**Consumers Only**														

2-18 y														
**Year**	**Mean ± SE**	**P**	**Mean ± SE**	**P**	**Mean ± SE**	**P**	**Mean ± SE**	**P**	**Mean ± SE**	**P**	**Mean ± SE**	**P**	**Mean ± SE**	**P**
**2003-04**	8.79 ± 0.34		7.38 ± 0.29		4.96 ± 0.23		2.50 ± 0.69		3.13 ± 0.32		2.18 ± 0.11		8.66 ± 0.13	
**2005-06**	8.11 ± 0.27	0.12	6.80 ± 0.25	0.14	4.52 ± 0.13	0.09	3.89 ± 0.27	0.06	2.67 ± 0.15	0.19	2.23 ± 0.09	0.71	9.00 ± 0.16	0.10
**2007-08**	8.12 ± 0.35	0.17	6.84 ± 0.47	0.33	5.08 ± 0.14	0.68	3.56 ± 0.45	0.20	3.18 ± 0.24	0.90	2.21 ± 0.13	0.85	8.90 ± 0.14	0.20
**2009-10**	7.35 ± 0.27	<0.01*	6.04 ± 0.21	<0.01*	4.90 ± 0.18	0.84	3.38 ± 0.26	0.23	3.88 ± 0.27	0.07	2.44 ± 0.08	0.07	8.44 ± 0.18	0.33
**2011-12**	6.76 ± 0.26	<0.01*	5.67 ± 0.28	<0.01*	4.39 ± 0.22	0.07	3.69 ± 0.26	0.11	5.27 ± 0.53	<0.01*	2.21 ± 0.16	0.86	8.33 ± 0.22	0.20
**2013-14**	6.96 ± 0.28	<0.01*	6.21 ± 0.33	0.01*	3.88 ± 0.17	<0.01*	4.76 ± 0.89	0.05	5.53 ± 1.28	0.07	2.02 ± 0.12	0.34	8.37 ± 0.25	0.31
**2015-16**	5.91 ± 0.19	<0.01*	5.50 ± 0.19	<0.01*	3.83 ± 0.14	<0.01*	3.42 ± 0.50	0.28	4.56 ± 0.23	<0.01*	2.02 ± 0.10	0.31	8.14 ± 0.21	0.04
**2017-18**	5.72 ± 0.26	<0.01*	5.38 ± 0.37	<0.01*	3.73 ± 0.20	<0.01*	3.46 ± 0.28	0.20	4.26 ± 0.31	0.01	2.14 ± 0.16	0.83	8.56 ± 0.22	0.72
**Trend**[1]	**Beta ± SE**	**P**	**Beta ± SE**	**P**	**Beta ± SE**	**P**	**Beta ± SE**	**P**	**Beta ± SE**	**P**	**Beta ± SE**	**P**	**Beta ± SE**	**P**
	-0.44 ± 0.05	<0.01	-0.28 ± 0.05	<0.01	-0.19 ± 0.03	<0.01	0.002 ± 0.07	0.98	0.29 ± 0.06	<0.01	-0.03 ± 0.02	0.19	-0.08 ± 0.03	0.01

*Significantly different from reference cycle (2003-04), p<0.01

[1]Linear trend across all cycles, statistically significant at p<0.01

Supplemental Table S3. Trends in added sugars intake (grams) from beverage sources and the rest of the diet among all children, adolescents and teens and consumers only from the pooled sample (NHANES 2003-2018, n=21,005).

**Food Group**	**Sweetened Beverages**		**Soft Drinks**		**Fruit Drinks**		**Sport and Energy Drinks**		**Coffee and Tea**		**Flavored Milk**		**Rest of the Diet**	
**All Individuals 2-8 y**														
**Year**	**Mean ± SE**	**P**	**Mean ± SE**	**P**	**Mean ± SE**	**P**	**Mean ± SE**	**P**	**Mean ± SE**	**P**	**Mean ± SE**	**P**	**Mean ± SE**	**P**
**2003-04**	25.83 ± 1.35		12.52 ± 1.23		13.25 ± 1.01		0.02 ± 0.01		1.99 ± 0.69		4.07 ± 0.48		41.57 ± 1.31	
**2005-06**	18.12 ± 0.94	<0.01*	7.29 ± 0.63	<0.01*	9.45 ± 0.64	<0.01*	1.18 ± 0.36	<0.01*	0.57 ± 0.12	0.04	3.25 ± 0.38	0.18	42.10 ± 1.26	0.77
**2007-08**	18.91 ± 0.79	<0.01*	7.47 ± 0.84	<0.01*	10.87 ± 0.53	0.04	0.50 ± 0.15	<0.01*	1.14 ± 0.31	0.26	4.00 ± 0.47	0.91	38.50 ± 1.08	0.07
**2009-10**	15.83 ± 0.63	<0.01*	5.40 ± 0.48	<0.01*	9.60 ± 0.61	<0.01*	0.72 ± 0.24	<0.01*	1.09 ± 0.14	0.20	4.28 ± 0.35	0.73	35.68 ± 1.05	<0.01*
**2011-12**	16.58 ± 1.00	<0.01*	6.51 ± 0.93	<0.01*	9.19 ± 0.61	<0.01*	0.84 ± 0.23	<0.01*	1.20 ± 0.19	0.27	3.95 ± 0.78	0.90	39.20 ± 1.30	0.20
**2013-14**	11.92 ± 1.03	<0.01*	3.86 ± 0.40	<0.01*	6.80 ± 0.88	<0.01*	1.12 ± 0.29	<0.01*	1.34 ± 0.49	0.44	2.80 ± 0.22	0.02	37.87 ± 1.70	0.09
**2015-16**	11.85 ± 0.89	<0.01*	4.26 ± 0.50	<0.01*	6.71 ± 0.48	<0.01*	0.77 ± 0.17	<0.01*	0.99 ± 0.27	0.18	2.56 ± 0.24	0.01	34.96 ± 0.92	<0.01*
**2017-18**	11.01 ± 1.01	<0.01*	4.36 ± 0.75	<0.01*	5.46 ± 0.49	<0.01*	1.06 ± 0.23	<0.01*	1.58 ± 0.51	0.64	3.21 ± 0.46	0.20	36.08 ± 1.32	<0.01*
**Trend**[1]	**Beta ± SE**	**P**	**Beta ± SE**	**P**	**Beta ± SE**	**P**	**Beta ± SE**	**P**	**Beta ± SE**	**P**	**Beta ± SE**	**P**	**Beta ± SE**	**P**
	-1.84 ± 0.17	<0.01	-0.97 ± 0.14	<0.01	-0.96 ± 0.11	<0.01	0.09 ± 0.03	0.01	-0.001 ± 0.08	0.99	-0.16 ± 0.06	0.01	-0.86 ± 0.20	<0.01
**All Individuals 9-18 y**														
**Year**	**Mean ± SE**	**P**	**Mean ± SE**	**P**	**Mean ± SE**	**P**	**Mean ± SE**	**P**	**Mean ± SE**	**P**	**Mean ± SE**	**P**	**Mean ± SE**	**P**
**2003-04**	50.00 ± 2.04		35.26 ± 1.50		14.67 ± 1.21		0.03 ± 0.02		2.66 ± 0.45		2.20 ± 0.20		47.15 ± 1.10	
**2005-06**	44.97 ± 3.88	0.25	31.31 ± 3.17	0.26	9.98 ± 0.71	<0.01*	3.55 ± 0.41	<0.01*	2.26 ± 0.45	0.53	2.29 ± 0.32	0.83	46.44 ± 0.85	0.61
**2007-08**	38.12 ± 2.47	<0.01*	25.22 ± 2.60	<0.01*	9.69 ± 0.48	<0.01*	3.08 ± 0.48	<0.01*	3.23 ± 0.63	0.46	2.05 ± 0.26	0.63	44.42 ± 1.27	0.11
**2009-10**	33.96 ± 1.64	<0.01*	20.73 ± 1.03	<0.01*	10.37 ± 0.97	0.01	2.76 ± 0.63	<0.01*	3.66 ± 0.74	0.25	3.09 ± 0.39	0.05	42.50 ± 0.90	<0.01*
**2011-12**	31.10 ± 1.09	<0.01*	18.15 ± 1.03	<0.01*	9.70 ± 0.45	<0.01*	3.19 ± 0.35	<0.01*	5.76 ± 0.95	<0.01*	2.12 ± 0.23	0.79	40.11 ± 1.68	<0.01*
**2013-14**	29.72 ± 2.12	<0.01*	18.54 ± 1.25	<0.01*	6.54 ± 0.46	<0.01*	4.48 ± 1.65	0.01	4.94 ± 0.77	0.01	1.98 ± 0.29	0.53	39.28 ± 1.76	<0.01*
**2015-16**	23.58 ± 1.44	<0.01*	15.32 ± 1.07	<0.01*	5.89 ± 0.49	<0.01*	2.14 ± 0.38	<0.01*	4.31 ± 0.73	0.06	2.07 ± 0.30	0.71	39.31 ± 1.82	<0.01*
**2017-18**	22.44 ± 1.64	<0.01*	13.53 ± 1.50	<0.01*	6.22 ± 0.64	<0.01*	2.27 ± 0.25	<0.01*	4.38 ± 0.45	0.01	1.60 ± 0.19	0.03	41.13 ± 1.23	<0.01*
**Trend**[1]	**Beta ± SE**	**P**	**Beta ± SE**	**P**	**Beta ± SE**	**P**	**Beta ± SE**	**P**	**Beta ± SE**	**P**	**Beta ± SE**	**P**	**Beta ± SE**	**P**
	-3.90 ± 0.35	<0.01	-3.02 ± 0.29	<0.01	-1.07 ± 0.13	<0.01	0.15 ± 0.08	0.05	0.35 ± 0.08	<0.01	-0.08 ± 0.04	0.04	-1.13 ± 0.20	<0.01
**All Individuals 2-18 y**														
**Year**	**Mean ± SE**	**P**	**Mean ± SE**	**P**	**Mean ± SE**	**P**	**Mean ± SE**	**P**	**Mean ± SE**	**P**	**Mean ± SE**	**P**	**Mean ± SE**	**P**

**2003-04**	40.38 ± 1.59		26.21 ± 1.33		14.10 ± 0.78		0.03 ± 0.01		2.39 ± 0.32		2.95 ± 0.23		44.93 ± 0.65	
**2005-06**	34.31 ± 2.33	0.03	21.78 ± 1.88	0.06	9.77 ± 0.48	<0.01*	2.61 ± 0.33	<0.01*	1.59 ± 0.26	0.06	2.67 ± 0.30	0.46	44.72 ± 0.84	0.84
**2007-08**	30.46 ± 1.51	<0.01*	18.14 ± 1.66	<0.01*	10.16 ± 0.34	<0.01*	2.05 ± 0.30	<0.01*	2.40 ± 0.45	0.99	2.82 ± 0.29	0.74	42.06 ± 0.87	0.01
**2009-10**	26.64 ± 1.18	<0.01*	14.54 ± 0.76	<0.01*	10.06 ± 0.74	<0.01*	1.94 ± 0.40	<0.01*	2.62 ± 0.46	0.69	3.57 ± 0.24	0.07	39.75 ± 0.65	<0.01*
**2011-12**	24.98 ± 0.92	<0.01*	13.25 ± 0.89	<0.01*	9.49 ± 0.38	<0.01*	2.20 ± 0.27	<0.01*	3.84 ± 0.56	0.03	2.89 ± 0.44	0.91	39.73 ± 1.22	<0.01*
**2013-14**	22.71 ± 1.48	<0.01*	12.76 ± 0.93	<0.01*	6.64 ± 0.44	<0.01*	3.16 ± 1.04	<0.01*	3.52 ± 0.62	0.11	2.30 ± 0.22	0.04	38.72 ± 1.56	<0.01*
**2015-16**	18.85 ± 1.07	<0.01*	10.86 ± 0.74	<0.01*	6.22 ± 0.37	<0.01*	1.58 ± 0.27	<0.01*	2.97 ± 0.53	0.36	2.27 ± 0.21	0.03	37.55 ± 1.32	<0.01*
**2017-18**	17.97 ± 1.00	<0.01*	9.95 ± 1.01	<0.01*	5.92 ± 0.46	<0.01*	1.80 ± 0.17	<0.01*	3.29 ± 0.45	0.11	2.23 ± 0.24	0.03	39.16 ± 0.99	<0.01*
**Trend[1]**	**Beta ± SE**	**P**	**Beta ± SE**	**P**	**Beta ± SE**	**P**	**Beta ± SE**	**P**	**Beta ± SE**	**P**	**Beta ± SE**	**P**	**Beta ± SE**	**P**
	-3.08 ± 0.23	<0.01	-2.21 ± 0.20	<0.01	-1.02 ± 0.08	<0.01	0.13 ± 0.05	0.01	0.21 ± 0.07	<0.01	-0.11 ± 0.04	<0.01	-1.02 ± 0.15	<0.01
**Consumers Only 2-8 y**														
**Year**	**Mean ± SE**	**P**	**Mean ± SE**	**P**	**Mean ± SE**	**P**	**Mean ± SE**	**P**	**Mean ± SE**	**P**	**Mean ± SE**	**P**	**Mean ± SE**	**P**
**2003-04**	31.44 ± 1.73		22.67 ± 1.63		22.87 ± 1.59		14.05 ± 3.56		15.32 ± 3.53		11.55 ± 1.14		41.57 ± 1.31	
**2005-06**	24.77 ± 1.09	<0.01*	17.34 ± 1.15	0.01	18.39 ± 0.86	0.01	13.42 ± 1.69	0.87	8.36 ± 0.88	0.06	10.54 ± 0.71	0.46	42.10 ± 1.26	0.77
**2007-08**	25.15 ± 0.68	<0.01*	17.72 ± 1.34	0.02	20.13 ± 0.84	0.13	7.96 ± 1.62	0.12	9.41 ± 1.10	0.11	11.30 ± 0.85	0.86	38.54 ± 1.08	0.08
**2009-10**	23.29 ± 0.80	<0.01*	16.04 ± 1.04	<0.01*	19.80 ± 0.84	0.09	11.86 ± 1.67	0.58	13.07 ± 0.85	0.54	11.83 ± 0.66	0.83	35.70 ± 1.05	<0.01*
**2011-12**	23.27 ± 1.31	<0.01*	17.10 ± 1.54	0.01	18.17 ± 1.10	0.02	11.35 ± 2.05	0.51	11.82 ± 1.54	0.36	11.37 ± 1.32	0.92	39.20 ± 1.30	0.20
**2013-14**	20.41 ± 1.09	<0.01*	15.18 ± 1.13	<0.01*	15.82 ± 0.98	<0.01*	12.33 ± 2.19	0.68	14.62 ± 3.13	0.88	9.39 ± 0.67	0.10	37.89 ± 1.70	0.09
**2015-16**	19.89 ± 0.80	<0.01*	16.98 ± 1.17	0.01	15.59 ± 0.97	<0.01*	11.78 ± 2.67	0.61	11.79 ± 1.88	0.38	9.66 ± 0.80	0.18	34.97 ± 0.92	<0.01*
**2017-18**	18.15 ± 1.39	<0.01*	15.48 ± 1.80	<0.01*	14.39 ± 0.86	<0.01*	11.66 ± 1.82	0.55	14.54 ± 2.18	0.85	9.95 ± 0.80	0.25	36.08 ± 1.32	<0.01*
**Trend[1]**	**Beta ± SE**	**P**	**Beta ± SE**	**P**	**Beta ± SE**	**P**	**Beta ± SE**	**P**	**Beta ± SE**	**P**	**Beta ± SE**	**P**	**Beta ± SE**	**P**
	-1.60 ± 0.22	<0.01	-0.81 ± 0.25	<0.01	-1.05 ± 0.19	<0.01	0.03 ± 0.38	0.93	0.19 ± 0.46	0.67	-0.26 ± 0.14	0.07	-0.86 ± 0.20	<0.01
**Consumers Only 9-18 y**														
**Year**	**Mean ± SE**	**P**	**Mean ± SE**	**P**	**Mean ± SE**	**P**	**Mean ± SE**	**P**	**Mean ± SE**	**P**	**Mean ± SE**	**P**	**Mean ± SE**	**P**
**2003-04**	55.61 ± 2.11		47.02 ± 2.00		28.79 ± 1.63		12.09 ± 2.64		16.21 ± 1.38		10.83 ± 0.58		47.15 ± 1.10	
**2005-06**	53.54 ± 3.75	0.63	46.00 ± 4.08	0.82	24.91 ± 1.01	0.04	23.50 ± 0.70	<0.01*	17.05 ± 1.67	0.70	11.57 ± 1.43	0.63	46.44 ± 0.85	0.61
**2007-08**	47.65 ± 2.49	0.02	41.69 ± 3.19	0.16	24.75 ± 0.96	0.03	21.65 ± 2.18	0.01	16.98 ± 1.83	0.74	8.98 ± 0.57	0.02	44.44 ± 1.27	0.11
**2009-10**	43.15 ± 1.98	<0.01*	35.25 ± 1.26	<0.01*	25.77 ± 1.36	0.16	19.66 ± 2.08	0.03	23.20 ± 3.32	0.05	10.81 ± 0.69	0.98	42.54 ± 0.90	<0.01*
**2011-12**	37.57 ± 1.38	<0.01*	31.76 ± 1.41	<0.01*	22.57 ± 1.38	<0.01*	20.42 ± 1.13	<0.01*	28.98 ± 2.64	<0.01*	9.51 ± 0.45	0.07	40.11 ± 1.68	<0.01*
**2013-14**	40.18 ± 3.07	<0.01*	36.20 ± 2.13	<0.01*	17.46 ± 0.68	<0.01*	30.35 ± 10.15	0.08	26.49 ± 4.45	0.03	8.92 ± 0.95	0.09	39.31 ± 1.76	<0.01*

**2015-16**	32.43 ± 1.70	<0.01*	30.27 ± 1.32	<0.01*	18.24 ± 1.32	<0.01*	18.69 ± 2.63	0.08	25.97 ± 2.05	<0.01*	8.83 ± 0.58	0.02	39.43 ± 1.85	<0.01*
**2017-18**	31.35 ± 2.16	<0.01*	29.99 ± 2.27	<0.01*	18.35 ± 1.47	<0.01*	18.16 ± 1.14	0.04	21.15 ± 1.72	0.03	8.05 ± 0.70	<0.01*	41.24 ± 1.25	<0.01*
**Trend[1]**	**Beta ± SE**	**P**	**Beta ± SE**	**P**	**Beta ± SE**	**P**	**Beta ± SE**	**P**	**Beta ± SE**	**P**	**Beta ± SE**	**P**	**Beta ± SE**	**P**
	-3.63 ± 0.38	<0.01	-2.67 ± 0.38	<0.01	-1.58 ± 0.22	<0.01	-0.30 ± 0.53	0.57	1.25 ± 0.29	<0.01	-0.41 ± 0.13	<0.01	-1.11 ± 0.20	<0.01
**Consumers Only<br>2-18 y**														
**Year**	**Mean ± SE**	**P**	**Mean ± SE**	**P**	**Mean ± SE**	**P**	**Mean ± SE**	**P**	**Mean ± SE**	**P**	**Mean ± SE**	**P**	**Mean ± SE**	**P**
**2003-04**	46.51 ± 1.79		39.05 ± 1.85		26.25 ± 1.06		12.53 ± 1.99		15.91 ± 1.60		11.21 ± 0.68		44.93 ± 0.65	
**2005-06**	43.06 ± 2.48	0.26	37.72 ± 2.92	0.70	21.93 ± 0.61	<0.01*	20.70 ± 0.61	<0.01*	14.85 ± 1.60	0.64	11.05 ± 0.72	0.87	44.72 ± 0.84	0.84
**2007-08**	39.01 ± 1.59	<0.01*	34.11 ± 2.38	0.10	22.54 ± 0.67	<0.01*	18.52 ± 2.36	0.05	14.73 ± 1.39	0.58	10.16 ± 0.56	0.23	42.09 ± 0.86	0.01
**2009-10**	35.82 ± 1.28	<0.01*	29.88 ± 0.95	<0.01*	23.09 ± 0.81	0.02	17.90 ± 1.73	0.04	20.54 ± 2.56	0.13	11.28 ± 0.34	0.93	39.78 ± 0.65	<0.01*
**2011-12**	32.06 ± 1.20	<0.01*	26.97 ± 1.23	<0.01*	20.54 ± 0.79	<0.01*	18.10 ± 1.28	0.02	24.32 ± 2.60	0.01	10.50 ± 0.87	0.52	39.73 ± 1.22	<0.01*
**2013-14**	33.48 ± 2.18	<0.01*	31.07 ± 1.95	<0.01*	16.76 ± 0.61	<0.01*	25.19 ± 7.22	0.09	23.62 ± 3.49	0.05	9.14 ± 0.65	0.03	38.75 ± 1.56	<0.01*
**2015-16**	27.96 ± 1.23	<0.01*	26.93 ± 0.98	<0.01*	16.98 ± 0.76	<0.01*	16.77 ± 2.24	0.16	22.36 ± 1.21	<0.01*	9.19 ± 0.42	0.01	37.63 ± 1.33	<0.01*
**2017-18**	26.70 ± 1.40	<0.01*	25.84 ± 1.77	<0.01*	16.69 ± 0.93	<0.01*	16.09 ± 0.90	0.11	19.49 ± 1.36	0.09	9.02 ± 0.61	0.02	39.22 ± 1.01	<0.01*
**Trend[1]**	**Beta ± SE**	**P**	**Beta ± SE**	**P**	**Beta ± SE**	**P**	**Beta ± SE**	**P**	**Beta ± SE**	**P**	**Beta ± SE**	**P**	**Beta ± SE**	**P**
	-2.82 ± 0.27	<0.01	-1.96 ± 0.31	<0.01	-1.34 ± 0.14	<0.01	-0.31 ± 0.39	0.43	1.04 ± 0.25	<0.01	-0.34 ± 0.10	<0.01	-1.01 ± 0.15	<0.01

*Significantly different from reference cycle (2003-04), p<0.01

[1]Linear trend across all cycles, statistically significant at p<0.01

Supplemental Table S4. Trends in % <EAR (>AI[1]) for selected micronutrients by beverage source of added sugars and the rest of the diet among children, adolescents and teens (2-18 y) from the pooled sample (NHANES 2003-2018, n=21,005); weighted n's are shown in the table.

**2-18 y**							
**Sweetened Beverages**							
	**Q1**[2] n=17,501,646 **% <EAR (SE)**	**Q2** n=17,351,739 **% <EAR (SE)**	**Q3** n=17,431,391 **% <EAR (SE)**	**Q4** n=17,421,108 **% <EAR (SE)**	**Quantile Trend**[3] **Beta (SE), P**	**Q1 vs. Q2,3,4**[4] **Beta (SE), P**	**Q2-Q4 Trend**[5] **Beta (SE), P**
**Calcium**	38.65 (1.49)	45.62 (1.31)	52.41 (1.51)	49.46 (1.47)	3.77 (1.75), 0.16	10.61 (4.14), 0.12	1.81 (2.83), 0.64
**Magnesium**	27.88 (1.15)	33.70 (1.24)	39.18 (1.18)	42.02 (1.03)	4.74 (0.51), 0.01	10.60 (5.17), 0.18	4.13 (0.77), 0.12
**Phosphorus**	13.54 (1.10)	16.48 (1.04)	18.17 (1.13)	12.03 (1.12)	-0.42 (1.54), 0.81	1.94 (3.99), 0.67	-2.31 (2.27), 0.49
**Vitamin A**	17.44 (1.41)	20.45 (1.30)	29.08 (1.33)	32.92 (1.62)	5.53 (0.75), 0.02§	10.32 (7.80), 0.32	6.18 (1.39), 0.14
**Vitamin C**	20.93 (1.40)	17.79 (1.48)	19.00 (1.58)	18.17 (1.74)	-0.67 (0.57), 0.36	-2.60 (0.75), 0.07	0.17 (0.59), 0.83
**Vitamin D**	88.86 (1.08)	91.38 (0.91)	93.99 (0.80)	94.17 (0.73)	1.82 (0.42), 0.05	4.38 (1.90), 0.15	1.37 (0.70), 0.30
**Choline**[1]	26.55 (1.27)	18.80 (1.18)	17.38 (1.04)	17.52 (0.96)	-2.82 (1.31), 0.16	-8.65 (0.93), 0.01§	-0.64 (0.46), 0.39
**Potassium**[1]	37.51 (1.63)	32.91 (1.76)	30.32 (1.60)	32.91 (1.39)	-1.53 (1.18), 0.32	-5.47 (1.85), 0.10	0.05 (1.51), 0.98
**Soft Drinks**							
	**Q1**[2] n=35,273,939 **% <EAR (SE)**	**Q2** n=11,356,667 **% <EAR (SE)**	**Q3** n=11,573,927 **% <EAR (SE)**	**Q4** n=11,501,350 **% <EAR (SE)**	**Quantile Trend**[3] **Beta (SE), P**	**Q1 vs. Q2,3,4**[4] **Beta (SE), P**	**Q2-Q4 Trend**[5] **Beta (SE), P**
**Calcium**	39.20 (0.98)	53.39 (1.66)	55.34 (1.96)	52.54 (1.76)	5.33 (2.45), 0.16	14.55 (1.18), 0.01†	-0.40 (1.36), 0.82
**Magnesium**	26.44 (0.73)	40.28 (1.71)	45.12 (1.21)	50.01 (1.32)	8.22 (1.40), 0.03§	18.61 (4.04), 0.04§	4.87 (0.02), 0.00†
**Phosphorus**	12.88 (0.68)	20.67 (1.30)	17.92 (1.54)	12.85 (1.47)	0.61 (1.90), 0.78	4.33 (3.28), 0.32	-3.90 (0.67), 0.11
**Vitamin A**	17.26 (0.94)	26.84 (1.74)	33.92 (1.75)	37.91 (2.05)	7.24 (0.78), 0.01§	15.52 (4.66), 0.08	5.55 (0.88), 0.10
**Vitamin C**	12.58 (0.93)	20.01 (1.75)	24.59 (2.14)	31.66 (2.56)	6.27 (0.32), 0.00†	12.74 (4.86), 0.12	5.81 (0.71), 0.08
**Vitamin D**	90.25 (0.67)	94.22 (0.96)	94.01 (0.97)	94.00 (0.94)	1.40 (0.63), 0.16	3.82 (0.10), 0.00†	-0.11 (0.06), 0.31
**Choline**[1]	25.05 (0.86)	15.16 (1.22)	13.69 (1.28)	15.52 (1.34)	-3.94 (1.72), 0.15	-10.28 (0.77), 0.01†	0.12 (0.96), 0.92
**Potassium**[1]	37.80 (1.15)	28.56 (1.86)	28.79 (1.94)	30.00 (1.87)	-3.06 (1.58), 0.19	-8.69 (0.64), 0.01†	0.71 (0.28), 0.24
**Fruit Drinks**							
	**Q1**[2] n=39,689,880 **% <EAR (SE)**	**Q2** n=9,979,489 **% <EAR (SE)**	**Q3** n=10,027,750 **% <EAR (SE)**	**Q4** n=10,008,765 **% <EAR (SE)**	**Quantile Trend**[3] **Beta (SE), P**	**Q1 vs. Q2,3,4**[4] **Beta (SE), P**	**Q2-Q4 Trend**[5] **Beta (SE), P**
**Calcium**	46.71 (1.05)	45.00 (2.01)	47.69 (1.55)	45.14 (1.71)	-0.29 (0.52), 0.63	-0.77 (1.22), 0.59	-0.04 (1.51), 0.99
**Magnesium**	39.63 (0.86)	29.11 (1.40)	31.33 (1.37)	29.66 (1.17)	-3.49 (1.48), 0.14	-9.58 (0.91), 0.01†	0.20 (1.12), 0.89
**Phosphorus**	16.07 (0.80)	15.42 (1.38)	13.97 (1.52)	10.97 (1.21)	-1.55 (0.31), 0.04	-2.80 (1.85), 0.27	-2.25 (0.45), 0.12
**Vitamin A**	26.68 (1.02)	20.83 (1.73)	21.39 (1.32)	24.71 (1.73)	-1.09 (1.23), 0.47	-4.20 (1.73), 0.14	2.00 (0.79), 0.24

**Vitamin C**	29.96 (1.22)	11.20 (1.69)	4.03 (1.46)	1.47 (1.02)	-10.25 (2.17), 0.04§	-24.79 (4.00), 0.03§	-4.77 (1.33), 0.17
**Vitamin D**	90.91 (0.63)	93.33 (0.92)	94.14 (0.94)	93.93 (0.95)	1.14 (0.35), 0.08	2.91 (0.33), 0.01	0.28 (0.30), 0.52
**Choline[1]**	19.90 (0.75)	20.49 (1.66)	20.07 (1.44)	20.34 (1.51)	0.14 (0.10), 0.32	0.40 (0.17), 0.14	-0.07 (0.20), 0.79
**Potassium[1]**	32.68 (1.10)	30.58 (1.90)	33.39 (2.01)	39.11 (1.83)	1.75 (1.04), 0.23	2.03 (3.56), 0.63	4.33 (0.84), 0.12
**Sport and Energy Drinks**							
	**Q1[2]**<br>n=62,719,734<br>**% <EAR (SE)**	**Q2**<br>n=2,248,682<br>**% <EAR (SE)**	**Q3**<br>n=2,358,945<br>**% <EAR (SE)**	**Q4**<br>n=2,378,523<br>**% <EAR (SE)**	**Quantile Trend[3]**<br>**Beta (SE), P**	**Q1 vs. Q2,3,4[4]**<br>**Beta (SE), P**	**Q2-Q4 Trend[5]**<br>**Beta (SE), P**
**Calcium**	46.17 (0.85)	56.69 (3.40)	47.72 (4.59)	41.41 (4.56)	-0.06 (2.34), 0.98	2.68 (4.64), 0.62	-7.66 (0.77), 0.06
**Magnesium**	34.53 (0.69)	48.67 (3.30)	45.87 (3.13)	43.22 (3.77)	4.48 (2.21), 0.18	11.47 (1.64), 0.02§	-2.73 (0.05), 0.01
**Phosphorus**	14.76 (0.63)	23.34 (3.55)	14.94 (3.58)	13.83 (2.73)	0.42 (1.71), 0.83	2.75 (3.17), 0.48	-4.82 (2.12), 0.26
**Vitamin A**	24.24 (0.80)	32.38 (3.15)	32.88 (4.47)	26.86 (5.27)	2.38 (1.65), 0.28	6.56 (1.99), 0.08	-2.71 (1.89), 0.39
**Vitamin C**	19.31 (0.86)	10.27 (5.03)	21.56 (3.86)	15.52 (6.57)	-1.10 (1.84), 0.61	-3.58 (3.45), 0.41	2.76 (5.03), 0.68
**Vitamin D**	92.00 (0.52)	94.51 (1.85)	92.17 (2.24)	91.76 (2.74)	0.15 (0.50), 0.79	0.86 (0.90), 0.44	-1.39 (0.56), 0.24
**Choline[1]**	20.98 (0.69)	13.20 (2.16)	14.64 (2.26)	14.96 (3.10)	-2.73 (1.12), 0.13	-6.73 (0.57), 0.01†	0.89 (0.32), 0.22
**Potassium[1]**	33.14 (1.11)	27.58 (3.25)	31.21 (3.78)	39.07 (4.40)	0.60 (1.60), 0.75	-0.71 (3.52), 0.86	5.71 (1.23), 0.13
**Coffee and Tea**							
	**Q1[2]**<br>n=59,622,348<br>**% <EAR (SE)**	**Q2**<br>n=3,317,496<br>**% <EAR (SE)**	**Q3**<br>n=3,406,193<br>**% <EAR (SE)**	**Q4**<br>n=3,359,846<br>**% <EAR (SE)**	**Quantile Trend[3]**<br>**Beta (SE), P**	**Q1 vs. Q2,3,4[4]**<br>**Beta (SE), P**	**Q2-Q4 Trend[5]**<br>**Beta (SE), P**
**Calcium**	44.58 (0.85)	62.76 (2.63)	55.44 (3.77)	53.26 (3.80)	4.79 (3.12), 0.26	12.95 (3.12), 0.05	-4.86 (1.51), 0.19
**Magnesium**	33.76 (0.72)	45.71 (2.51)	44.96 (2.37)	46.90 (3.22)	5.31 (1.54), 0.08	12.03 (0.58), 0.00†	0.53 (0.79), 0.62
**Phosphorus**	14.23 (0.68)	23.12 (2.22)	19.06 (2.53)	16.01 (3.35)	1.76 (1.71), 0.41	5.47 (2.20), 0.13	-3.58 (0.30), 0.05
**Vitamin A**	23.21 (0.85)	33.94 (3.54)	32.72 (3.63)	38.89 (3.72)	5.48 (1.19), 0.04§	11.72 (1.94), 0.03§	2.31 (2.17), 0.48
**Vitamin C**	17.03 (0.92)	28.35 (3.72)	27.66 (4.54)	36.95 (4.57)	6.56 (1.17), 0.03§	13.54 (3.07), 0.05§	4.07 (2.93), 0.40
**Vitamin D**	91.47 (0.56)	96.73 (0.97)	95.76 (1.21)	93.74 (1.92)	1.52 (0.96), 0.26	4.07 (0.92), 0.05	-1.47 (0.31), 0.13
**Choline[1]**	21.15 (0.67)	13.94 (1.69)	16.12 (2.21)	13.77 (2.22)	-2.80 (0.96), 0.10	-6.52 (0.82), 0.02§	-0.01 (1.33), 0.99
**Potassium[1]**	33.33 (0.96)	29.22 (3.06)	33.41 (3.03)	41.05 (3.88)	1.29 (1.48), 0.47	0.70 (3.63), 0.86	5.84 (1.02), 0.11
**Flavored Milk**							
	**Q1[2]**<br>n=51,222,508<br>**% <EAR (SE)**	**Q2**<br>n=5,797,437<br>**% <EAR (SE)**	**Q3**<br>n=6,342,564<br>**% <EAR (SE)**	**Q4**<br>n=6,343,373<br>**% <EAR (SE)**	**Quantile Trend[3]**<br>**Beta (SE), P**	**Q1 vs. Q2,3,4[4]**<br>**Beta (SE), P**	**Q2-Q4 Trend[5]**<br>**Beta (SE), P**
**Calcium**	52.59 (0.94)	38.64 (2.97)	30.18 (2.51)	10.15 (2.39)	-13.30 (1.07), 0.01†	-26.06 (9.76), 0.12	-14.21 (3.39), 0.15
**Magnesium**	40.50 (0.79)	22.34 (1.95)	23.04 (1.74)	18.56 (1.67)	-8.20 (2.17), 0.06	-19.14 (1.61), 0.01†	-1.87 (1.52), 0.43
**Phosphorus**	18.87 (0.70)	7.68 (1.32)	5.03 (1.40)	0.61 (0.36)	-6.55 (1.03), 0.02§	-14.38 (2.38), 0.03	-3.53 (0.52), 0.09

**Vitamin A**	30.00 (0.90)	10.85 (2.58)	11.02 (1.85)	6.74 (1.26)	-8.75 (2.29), 0.06	-20.42 (1.62), 0.01†	-2.05 (1.30), 0.36
**Vitamin C**	20.72 (1.07)	11.92 (1.66)	15.25 (1.99)	16.24 (1.85)	-2.17 (1.47), 0.28	-6.26 (1.52), 0.05	2.17 (0.69), 0.20
**Vitamin D**	93.44 (0.47)	98.77 (0.83)	93.45 (2.03)	84.90 (4.14)	-1.68 (1.79), 0.45	-0.98 (4.67), 0.85	-6.92 (0.95), 0.09
**Choline**[1]	18.20 (0.71)	17.85 (1.63)	22.75 (2.45)	34.72 (2.19)	4.29 (1.53), 0.11	6.58 (5.77), 0.37	8.38 (2.08), 0.16
**Potassium**[1]	29.90 (0.96)	31.52 (2.36)	40.04 (2.67)	62.96 (3.11)	8.91 (2.61), 0.08	14.70 (10.85), 0.31	15.68 (4.22), 0.17

**Rest of the Diet**					
**Added Sugars** (% kcal)	**Q1** n=17,415,441 ≤5.19	**Q2** n=17,427,804 >5.19 to ≤7.95	**Q3** n=17,414,410 >7.95 to ≤11.15	**Q4** n=17,448,227 >11.15	**Quartile Trend**[6]
	**% <EAR (SE)**	**% <EAR (SE)**	**% <EAR (SE)**	**% <EAR (SE)**	**Beta (SE), P**
**Calcium**	62.10 (1.25)	46.58 (1.34)	43.28 (1.72)	30.43 (1.41)	-9.91 (1.58), 0.02§
**Magnesium**	49.74 (1.19)	36.71 (1.09)	31.88 (1.17)	21.35 (1.08)	-9.07 (1.05), 0.01§
**Phosphorus**	31.14 (1.41)	14.70 (1.09)	10.37 (1.01)	4.13 (0.55)	-8.72 (1.90), 0.04§
**Vitamin A**	46.41 (1.39)	24.15 (1.44)	17.63 (1.66)	7.25 (1.16)	-12.62 (2.34), 0.03§
**Vitamin C**	26.58 (1.63)	19.83 (1.42)	16.86 (1.61)	12.55 (1.37)	-4.55 (0.53), 0.01
**Vitamin D**	95.05 (0.55)	93.57 (0.99)	92.11 (0.81)	88.63 (1.02)	-2.04 (0.34), 0.03
**Choline**[1]	17.22 (0.96)	19.03 (1.19)	19.66 (1.23)	23.01 (1.33)	1.78 (0.35), 0.04
**Potassium**[1]	19.33 (1.38)	30.64 (1.68)	34.04 (1.53)	49.95 (1.77)	9.49 (1.56), 0.03§

EAR, estimated average requirement; AI, adequate intake

[1]>AI for choline and potassium

[2]Quantile 1 represents non-consumers; the remaining sample of those reporting intake (consumers) was divided into tertiles (quantiles 2, 3 and 4)

[3]From regression analysis among all individuals, test for trend

[4]From regression analysis among all individuals, test for differences between non-consumers (Q1) and consumers (Q2,3,4)

[5]From regression analysis among consumers (Q2-Q4), test for trend

[6]From regression analysis, test for trend

†Statistically significant at $p<0.01$; §Statistically significant at $p<0.05$

Intakes of added sugars with a focus on beverages and the associations with micronutrient adequacy in US children, adolescents and teens (NHANES 2003-2018), Ricciuto, L., et al.

A

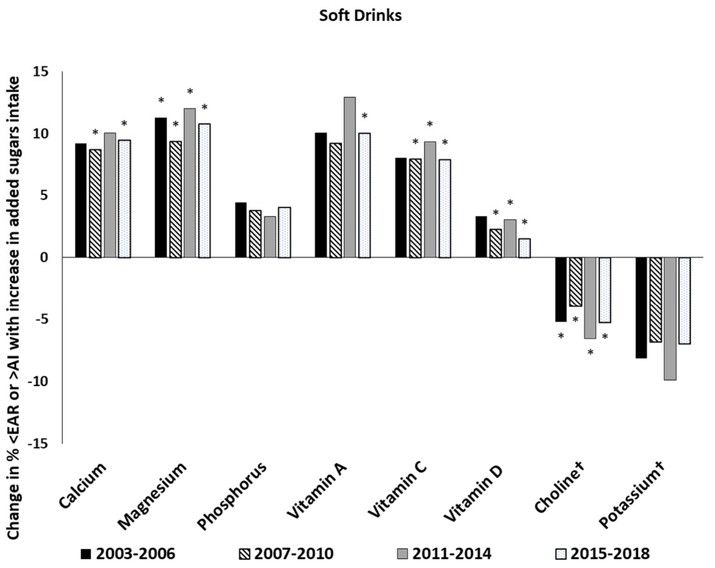

B

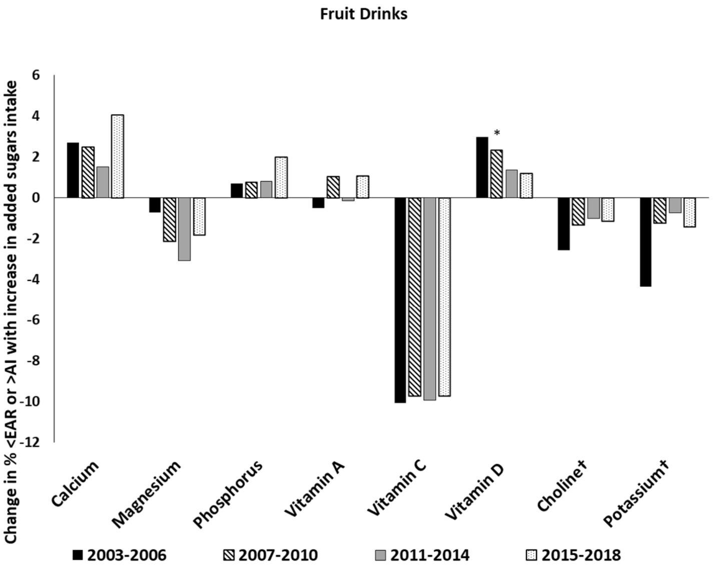

Intakes of added sugars with a focus on beverages and the associations with micronutrient adequacy in US children, adolescents and teens (NHANES 2003-2018), Ricciuto, L., et al.

C

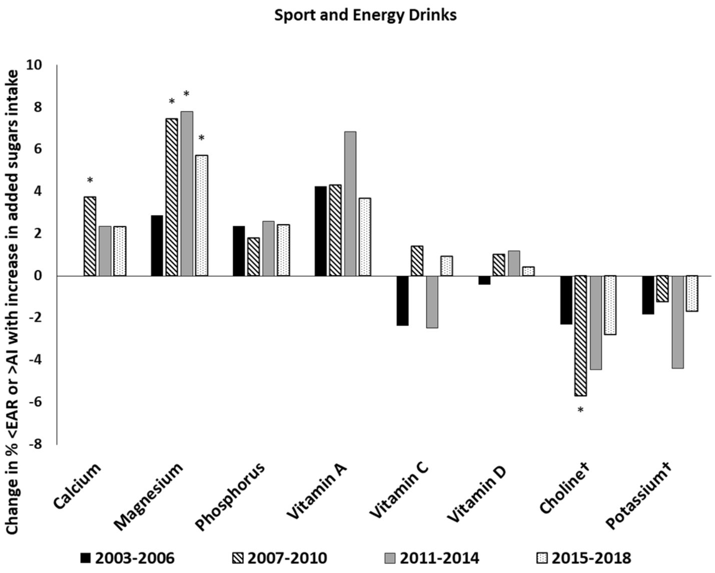

D

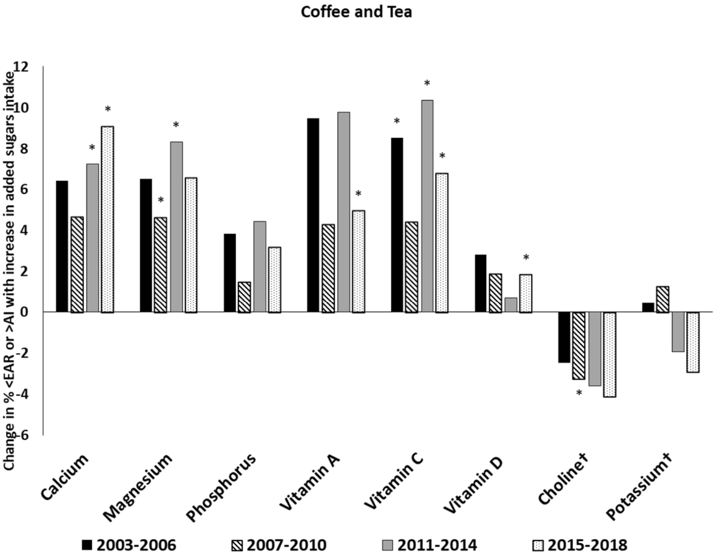

Supplemental Figure S2. Change in % <EAR (>AI[†]) in association with increased added sugars intake from select beverage sources: **A.** soft drinks and **B.** fruit drinks, by NHANES 4-y cycles, among children, adolescents and teens (2-18 y). **C.** sport and energy drinks and **D.** coffee and tea, by NHANES 4-y cycles, among children, adolescents and teens (2-18 y).

EAR, estimated average requirement; AI, adequate intake

[†]>AI for choline and potassium

*Statistically significant at $p<0.05$; from regression analysis, test for trend across quantiles of added sugars intake.

Supplemental Table S5. Association between added sugars intake from select beverages and % <EAR (>AI[1]) among children, adolescents and teens (2-18 y) by NHANES 4-y cycles.

	**NHANES 2003-2006**	**NHANES 2007-2010**	**NHANES 2011-2014**	**NHANES 2015-2018**
	**Soft Drinks**			
**Nutrient**	**Beta (SE), P[2]**	**Beta (SE), P**	**Beta (SE), P**	**Beta (SE), P**
**Calcium**	9.18 (3.00), 0.09	8.71 (1.75), 0.04*	10.02 (2.60), 0.06	9.43 (2.19), 0.05*
**Magnesium**	11.26 (2.41), 0.04*	9.35 (1.23), 0.02*	11.98 (2.18), 0.03*	10.74 (1.49), 0.02*
**Phosphorus**	4.42 (2.61), 0.23	3.76 (1.47), 0.12	3.32 (2.16), 0.26	4.05 (3.15), 0.33
**Vitamin A**	10.05 (3.26), 0.09	9.20 (2.38), 0.06	12.93 (4.93), 0.12	10.01 (1.32), 0.02*
**Vitamin C**	8.05 (3.32), 0.14	7.94 (1.17), 0.02*	9.34 (1.83), 0.04*	7.89 (1.16), 0.02*
**Vitamin D**	3.35 (0.81), 0.05	2.25 (0.43), 0.03*	3.04 (0.45), 0.02*	1.49 (0.14), 0.01*
**Choline[1]**	-5.22 (1.02), 0.04*	-3.94 (0.52), 0.02*	-6.56 (1.19), 0.03*	-5.26 (0.24), 0.00*
**Potassium[1]**	-8.14 (4.12), 0.19	-6.84 (2.15), 0.09	-9.90 (3.55), 0.11	-6.98 (3.60), 0.19
	**Fruit Drinks**			
**Nutrient**	**Beta (SE), P**	**Beta (SE), P**	**Beta (SE), P**	**Beta (SE), P**
**Calcium**	2.72 (3.10), 0.47	2.48 (3.66), 0.57	1.53 (3.21), 0.68	4.05 (3.45), 0.36
**Magnesium**	-0.71 (4.18), 0.88	-2.12 (2.90), 0.54	-3.06 (3.25), 0.45	-1.83 (3.73), 0.67
**Phosphorus**	0.70 (2.54), 0.81	0.76 (1.86), 0.72	0.81 (2.73), 0.79	2.01 (3.28), 0.60
**Vitamin A**	-0.50 (2.86), 0.88	1.03 (2.50), 0.72	-0.13 (4.20), 0.98	1.08 (3.32), 0.78
**Vitamin C**	-10.07 (2.65), 0.06	-9.73 (2.46), 0.06	-9.94 (2.88), 0.07	-9.72 (3.39), 0.10
**Vitamin D**	2.98 (1.54), 0.19	2.33 (0.17), 0.01*	1.36 (0.38), 0.07	1.19 (0.40), 0.10
**Choline[1]**	-2.55 (3.30), 0.52	-1.32 (1.50), 0.47	-1.01 (0.33), 0.09	-1.16 (2.32), 0.67
**Potassium[1]**	-4.35 (4.61), 0.44	-1.24 (2.39), 0.66	-0.73 (1.72), 0.71	-1.42 (1.89), 0.53
	**Sport and Energy Drinks**			
**Nutrient**	**Beta (SE), P**	**Beta (SE), P**	**Beta (SE), P**	**Beta (SE), P**
**Calcium**	0.00 (7.41), 1.00	3.72 (0.80), 0.04*	2.35 (2.25), 0.41	2.33 (3.81), 0.60
**Magnesium**	2.86 (3.75), 0.53	7.43 (1.24), 0.03*	7.79 (1.66), 0.04*	5.71 (1.07), 0.03*
**Phosphorus**	2.37 (3.66), 0.58	1.80 (2.23), 0.50	2.59 (4.20), 0.60	2.42 (3.32), 0.54
**Vitamin A**	4.23 (3.88), 0.39	4.29 (3.51), 0.35	6.83 (1.60), 0.05	3.66 (2.89), 0.33
**Vitamin C**	-2.39 (4.51), 0.65	1.41 (3.31), 0.71	-2.50 (1.02), 0.13	0.92 (5.40), 0.88
**Vitamin D**	-0.42 (4.03), 0.93	1.01 (2.22), 0.69	1.19 (0.82), 0.28	0.42 (1.51), 0.81
**Choline[1]**	-2.31 (2.82), 0.50	-5.71 (0.28), 0.00*	-4.45 (2.83), 0.26	-2.81 (1.23), 0.15
**Potassium[1]**	-1.84 (6.36), 0.80	-1.23 (2.53), 0.67	-4.40 (2.88), 0.27	-1.71 (5.19), 0.77
	**Coffee and Tea**			
**Nutrient**	**Beta (SE), P**	**Beta (SE), P**	**Beta (SE), P**	**Beta (SE), P**
**Calcium**	6.42 (2.42), 0.12	4.67 (2.41), 0.19	7.22 (1.57), 0.04*	9.09 (1.05), 0.01*
**Magnesium**	6.52 (2.62), 0.13	4.64 (0.87), 0.03*	8.32 (1.58), 0.03*	6.56 (1.62), 0.06
**Phosphorus**	3.83 (2.34), 0.24	1.48 (3.59), 0.72	4.43 (2.06), 0.16	3.20 (1.31), 0.13
**Vitamin A**	9.46 (2.90), 0.08	4.29 (3.27), 0.32	9.77 (2.29), 0.05	4.96 (0.44), 0.01*
**Vitamin C**	8.52 (1.90), 0.05*	4.41 (2.09), 0.17	10.35 (1.33), 0.02*	6.80 (0.71), 0.01*

**Vitamin D**	2.81 (1.89), 0.27	1.90 (1.68), 0.38	0.69 (0.82), 0.49	1.86 (0.33), 0.03*
**Choline**[1]	-2.50 (3.50), 0.55	-3.25 (0.19), 0.00*	-3.61 (1.54), 0.14	-4.11 (1.44), 0.10
**Potassium**[1]	0.44 (4.31), 0.93	1.29 (2.22), 0.62	-1.95 (3.50), 0.63	-2.92 (2.90), 0.42

EAR, estimated average requirement; AI, adequate intake

[1]>AI for choline and potassium

[2]Beta represents the regression coefficient (change) in percentage of the population <EAR (>AI) across quantiles of added sugars from the select beverage, testing hypothesis that regression coefficient (slope) equals 0

*Statistically significant at $p<0.05$

Supplemental Table S6. Quartiles of added sugars intake (% kcal) from the rest of the diet and trends in % <EAR (>AI[1]) for selected micronutrients among children, adolescents and teens from the pooled sample (NHANES 2003-2018, n=21,005); weighted n's are shown in the table.

**2-8 y (n=8,599)**					
**Added Sugars** (% kcal)	**Q1** n=6,974,607 <5.79	**Q2** n=6,993,758 5.79 to ≤8.46	**Q3** n=6,974,352 >8.46 to ≤11.62	**Q4** n=6,995,657 >11.62	**Quartile Trend**[2]
	**% <EAR* (SE)**	**% <EAR* (SE)**	**% <EAR* (SE)**	**% <EAR* (SE)**	**Beta (SE), P**
**Calcium**	24.96 (1.48)	17.89 (1.46)	18.55 (1.91)	13.80 (1.42)	-3.34 (1.00), 0.08
**Magnesium**	1.52 (0.33)	0.20 (0.12)	0.15 (0.07)	0.07 (0.04)	-0.46 (0.22), 0.17
**Phosphorus**	0.21 (0.09)	0.02 (0.02)	0.00 (0.00)	0.00 (0.00)	-0.07 (0.03), 0.17
**Vitamin A**	4.94 (0.81)	1.35 (0.49)	1.66 (0.52)	0.44 (0.24)	-1.37 (0.54), 0.13
**Vitamin C**	0.90 (0.32)	0.91 (0.30)	0.96 (0.34)	0.83 (0.31)	-0.02 (0.03), 0.61
**Vitamin D**	89.27 (1.08)	89.58 (1.48)	89.59 (1.16)	87.73 (1.30)	-0.42 (0.35), 0.35
**Choline**[1]	41.75 (2.16)	43.35 (2.15)	42.84 (2.14)	47.67 (2.40)	1.66 (0.70), 0.14
**Potassium**[1]	33.37 (1.92)	38.63 (1.99)	38.67 (2.13)	48.07 (2.13)	4.35 (1.18), 0.07
**9-18 y (n=12,406)**					
**Added Sugars** (% kcal)	**Q1** n=10,440,833 <4.78	**Q2** n=10,434,047 4.78 to ≤7.57	**Q3** n=10,440,058 >7.57 to ≤10.87	**Q4** n=10,452,571 >10.87	**Quartile Trend**[2]
	**% <EAR (SE)**	**% <EAR (SE)**	**% <EAR (SE)**	**% <EAR (SE)**	**Beta (SE), P**
**Calcium**	87.09 (1.50)	65.71 (1.96)	59.84 (2.48)	41.42 (1.89)	-14.39 (2.00), 0.02§
**Magnesium**	82.03 (1.20)	61.30 (1.47)	52.94 (1.75)	35.67 (1.76)	-14.84 (1.58), 0.01§
**Phosphorus**	51.38 (2.06)	24.29 (1.79)	17.37 (1.64)	7.00 (0.88)	-14.28 (3.12), 0.04§
**Vitamin A**	74.24 (1.68)	39.79 (2.29)	28.47 (2.63)	11.83 (1.83)	-20.16 (3.45), 0.03§
**Vitamin C**	43.89 (2.40)	32.53 (2.18)	27.58 (2.38)	20.39 (2.13)	-7.62 (0.90), 0.01§
**Vitamin D**	98.82 (0.25)	96.11 (0.77)	93.85 (0.79)	89.30 (1.12)	-3.06 (0.34), 0.01
**Choline**[1]	0.98 (0.31)	2.95 (0.80)	4.56 (0.94)	6.04 (1.17)	1.69 (0.08), 0.00†
**Potassium**[1]	9.80 (1.24)	25.17 (1.80)	30.83 (1.95)	50.81 (2.16)	12.86 (1.77), 0.02§

EAR, estimated average requirement; AI, adequate intake

[1]>AI for choline and potassium

[2]From regression analysis, test for trend

†Statistically significant at p<0.01; §Statistically significant at p<0.05
